# NLRP3 Inflammasome Activation Modulates Neutrophil Extracellular Trap Formation and Aggravates Airway Inflammation in Bronchiectasis

**DOI:** 10.34133/research.0958

**Published:** 2025-10-17

**Authors:** Zhao-ming Chen, Jia-hui He, Rui-di Tang, Jun-qing Yue, Xiao-xian Zhang, Zhen-feng He, Shao-qiang Li, Hui-min Li, Xiao-fen Zhang, Lai-jian Cen, Cui-xia Pan, Ming-xin Shi, Sheng-zhu Lin, Shu-jun Guo, Wei-jie Guan

**Affiliations:** ^1^ State Key Laboratory of Respiratory Disease, National Clinical Research Center for Respiratory Disease, National Center for Respiratory Medicine, Joint International Research Laboratory of Respiratory Health, Guangdong Basic Research Center of Excellence for Respiratory Medicine, Department of Allergy and Clinical Immunology, Department of Respiratory and Critical Care Medicine, Guangzhou Institute of Respiratory Health, the First Affiliated Hospital of Guangzhou Medical University, Guangzhou, 510163, P. R. China.; ^2^ Division of Respiratory Medicine and Gastroenterology, University of Dundee, Ninewells Hospital and Medical School, Dundee, UK.; ^3^ Guangzhou National Laboratory, Guangzhou, 510005, P. R.China.; ^4^ China-Portugal Artificial Intelligence and Public Health Technologies Joint Laboratory, Guangdong-Hong Kong-Macao Joint Laboratory of Respiratory Infectious Diseases, Guangdong Provincial Key Laboratory of Respiratory Disease Research, Guangzhou Medical University, Guangzhou, 510163, P. R. China.; ^5^ Shanxi Bethune Hospital, Taiyuan, 050081, P. R. China.

## Abstract

Neutrophilic inflammation and *Pseudomonas aeruginosa* infection have been implicated in the pathogenesis and progression of bronchiectasis. However, the mechanisms underlying the inflammasome activation and neutrophil extracellular traps (NETs) formation, which are triggered by lipopolysaccharide (LPS), thereby fostering relentless neutrophilic inflammation and airway destruction, remain poorly understood. Here, we investigated how nucleotide-binding oligomerization domain-like receptor protein 3 (NLRP3) inflammasome activation modulates NET formation, epithelial inflammation, and injury in bronchiectasis. Compared with those in the stable state, interleukin-1β levels in the exacerbation state were greater and were correlated with blood neutrophil counts. LPS triggered neutrophil NLRP3 inflammasome activation and induced NET formation. At exacerbation, caspase-1 p20, DNA-myeloperoxidase, and DNA-neutrophil elastase expression levels were increased compared with those in the stable state. LPS triggered the first signal, nuclear factor-κB, for NLRP3 inflammasome activation within the airway epithelium, leading to neutrophil chemotactic factor release. NETs served as the secondary signal for thorough NLRP3 inflammasome activation. Acting synergistically with LPS, NETs amplified epithelial inflammation, up-regulating mucin 5AC expression while suppressing zona occludens-1 and α-tubulin expression. Both MCC950 and Z-VAD-FMK inhibited NLRP3 inflammasome activation, whereas GSK484 and LDC7559 suppressed NET formation. Collectively, NLRP3 inflammasome activation facilitates NET formation in bronchiectasis, aggravating inflammation and eliciting injury to epithelial cells.

## Introduction

Bronchiectasis is a chronic structural lung disease characterized by recurrent airway infections and mucopurulent or purulent sputum production [1–3], both of which are associated with exuberant neutrophilic airway inflammation [4,5]. However, the mechanisms are poorly understood.

Inflammasomes regulate inflammatory responses against microorganisms [6]. The nucleotide-binding oligomerization domain-like receptor protein 3 (NLRP3) inflammasome is a multiprotein complex ubiquitously expressed in immune and epithelial cells [7]. By recognizing damage- or pathogen-associated molecular patterns, the NLRP3 inflammasome activates both caspase-1 and gasdermin D (GSDMD), promoting interleukin (IL)-1β and IL-18 secretion, further leading to recruitment and activation of immune cells [8]. Increased NLRP3 inflammasome activity has been implicated in chronic obstructive pulmonary disease (COPD) and steroid-resistant asthma [9–11]. Bronchiectasis patients with high sputum IL-1β levels reportedly exhibit greater disease severity [12]. However, how inflammasome activation drives NET release to aggravate airway inflammatory damage remains unclear. Previous findings have been predominantly based on immortalized epithelial cell lines or human monocyte leukemia cell lines (e.g., THP-1). Systematic investigations of the expression patterns of inflammasomes in neutrophils and airway epithelial cells from bronchiectasis patients remain lacking, and their precise functional role in disease pathogenesis has yet to be elucidated.

The transition of neutrophils from the resting state to the activated state is key to increasing neutrophilic inflammation. Activated neutrophils eliminate pathogens by releasing neutrophil extracellular traps (NETs) that contain depolymerized chromatin and antimicrobial proteins [13]. Bronchiectasis patients reportedly exhibit abundant NET-related proteins within sputum, which are correlated with bronchiectasis severity, quality of life, and the future risk of hospitalization and death [14]. The NLRP3–GSDMD axis is a canonical rapid and reversible inflammatory activation system located within neutrophils that modulates neutrophil activation, protease release, phagocytosis, and bacterial killing. Under sterile stimulation, activation of the NLRP3 inflammasome in neutrophils leads to IL-1β release, which can induce pyroptosis or the formation of NETs. Upon inflammasome activation, GSDMD represents a key mediator of NET generation in neutrophils [15–17]; other studies have reported that GSDMD is dispensable for PMA-induced NETosis, indicating that NET formation can also occur independently of inflammasome activation [18,19]. How NLRP3 inflammasome activation precipitates NET formation in bronchiectasis merits investigation.

NETs contain proteinases such as myeloperoxidase (MPO), neutrophil elastase (NE), and metalloproteinases that predispose patients to airway destruction [20]. NE may increase susceptibility to bacterial infections [21], which promote neutrophil migration in bronchiectasis [22]. *Pseudomonas aeruginosa* (PsA), the bacterium most frequently isolated from the airways of bronchiectasis patients, is closely associated with disease progression and exacerbation [23,24]. Its lipopolysaccharide (LPS) is a key virulence factor. Patients with PsA infection or colonization experience more frequent acute exacerbations (AEs), more rapid lung function decline, and higher mortality rates [25,26]. However, intratracheal instillation of pathogenic bacteria (e.g., PsA) can elicit transient airway infection, suggesting a role of NETs in inducing durable inflammation and a synergistic effect of pathogens and NETs in eliciting epithelial destruction. Indeed, NETs can activate the NLRP3 inflammasome in glomerular endothelial cells, resulting in cellular dysfunction [27], and induce pyroptosis in macrophages in murine models of acute lung injury [28]. Existing drugs targeting NETs have certain limitations. Multiple clinical trials have demonstrated that single DNase treatments have limited clinical benefit and NE inhibitors only target NE protease activity, but do not actually cover the abundant proteases of NETs [29]. Therefore, further understanding the effect of NLRP3 inflammasome activation in neutrophil may provide insight into pathology and identify new therapeutic targets for the clinical treatment of neutrophilic inflammatory reactions in bronchiectasis.

The aim of this study was to determine whether NETs can activate the airway epithelial inflammasome and further amplify inflammation in conjunction with PsA LPS in bronchiectasis. We clarified the interplay between neutrophil NLRP3 inflammasome activation and NET formation, testing the hypothesis that NETs could activate airway epithelial inflammasome and further amplify the inflammation in conjunction with PsA LPS in bronchiectasis. Our findings may provide greater insights into the pathophysiology and treatment of neutrophilic inflammation in bronchiectasis.

## Results

### Sputum IL-1β levels were elevated at exacerbation and correlated with blood neutrophil counts and C-reactive protein

**Fig. 1.**
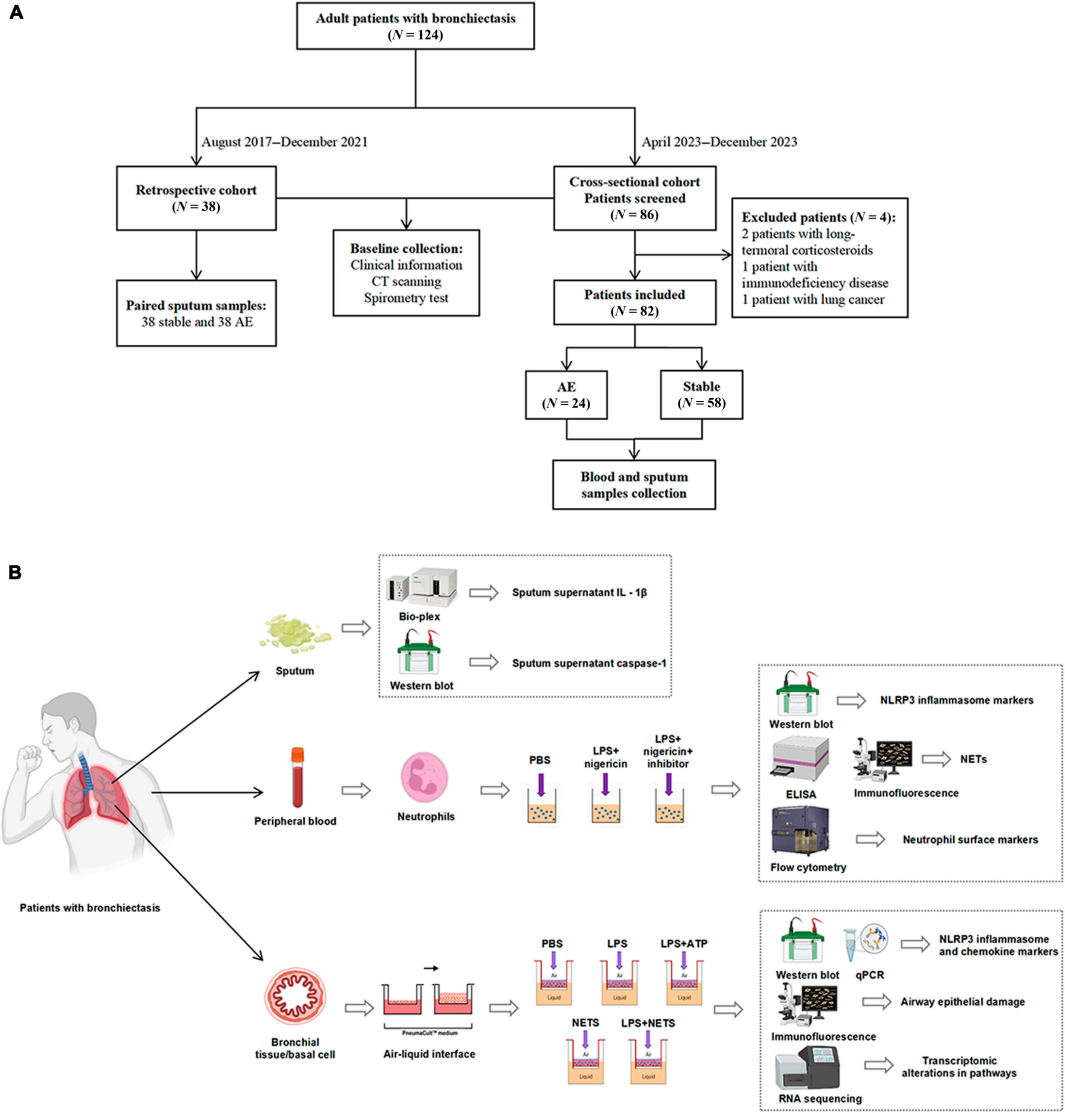
Flowchart of patient inclusion and study design. (A) Flowchart of patient inclusion. (B) Study design. Sputum samples were collected from patients, and the supernatants were analyzed for IL-1β using multiplex immunoassay kits. The presence of the cellular protein caspase-1 was determined via Western blot analysis. Neutrophils were isolated from the patients’ blood, and Western blotting was used to detect inflammasome markers, whereas flow cytometry was used to identify neutrophil surface markers. Additionally, ELISA and fluorescence assays were conducted to detect neutrophil extracellular traps (NETs). Bronchial epithelial cells were obtained from the bronchial tissues of patients with bronchiectasis and cultured using the air–liquid interface method. Western blotting and quantitative PCR were then used to identify inflammasome and chemokine markers. Pathological and fluorescence assays were applied to evaluate airway epithelial damage, and transcriptomic analysis was performed to identify alterations in associated pathways

**Fig. 2.**
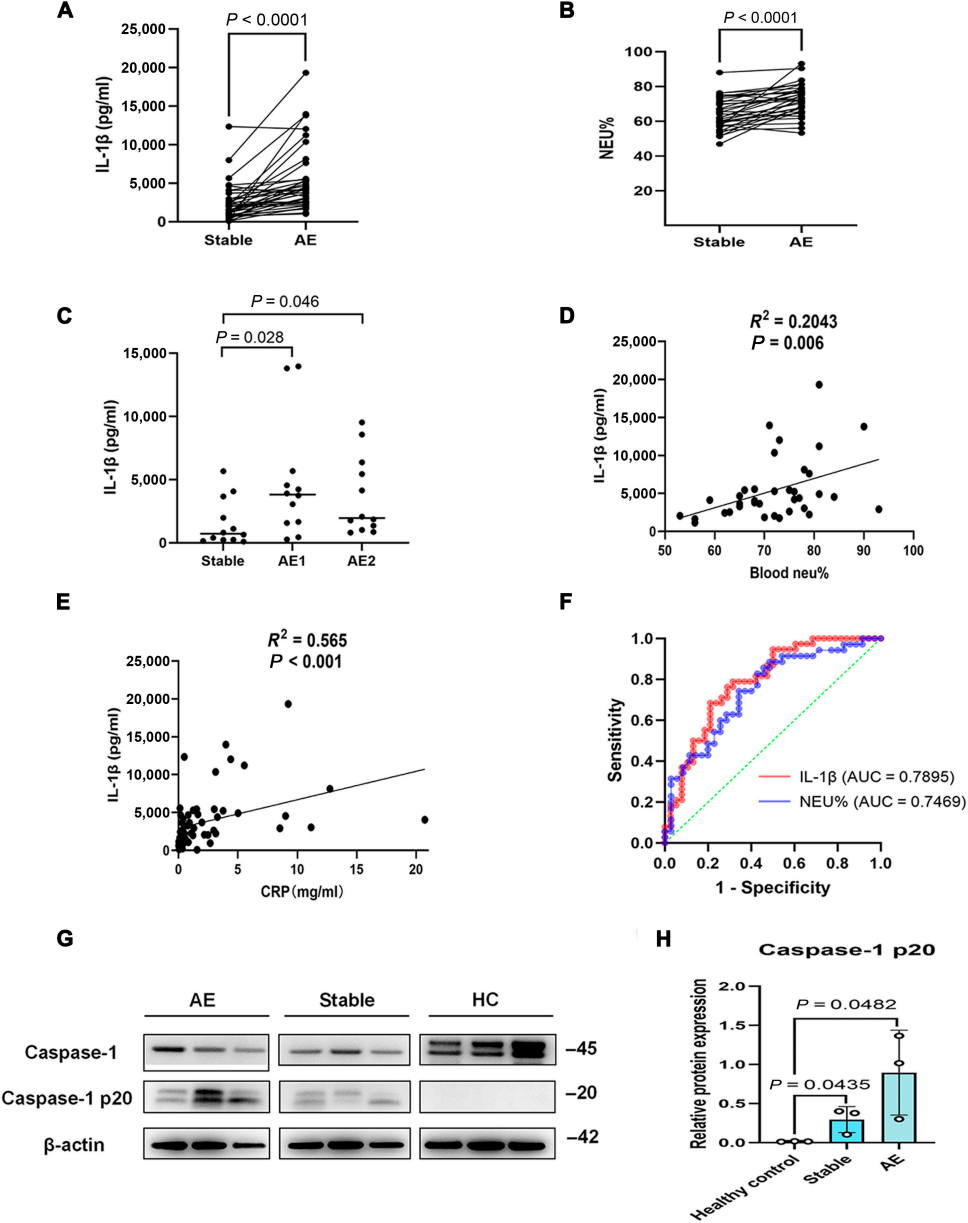
The expression levels of IL-1β in sputum and neutrophil percentages in peripheral blood of bronchiectasis patients at different clinical stages. (A) Levels of IL-1β in sputum and (B) percentages of neutrophils (NEU%) in the peripheral blood of patients with onset of bronchiectasis at AE were significantly elevated (*n* = 38). (C) In 12 of 38 patients, sputum IL-1β concentrations were elevated at both discrete acute exacerbations (AEs) compared with the stable-state baseline. (D) Association between IL-1β (combining stable state and AE samples) and NEU% in patients with bronchiectasis. (E) Association between IL-1β (combining stable state and AE samples) and CRP in patients with bronchiectasis. (F) ROC curves for the ability of sputum IL-1β and blood NEU% to distinguish between patients at AE and stable state. (G) Western blot analyses of caspase-1, caspase-1 p20, and the endogenous control β-actin in the sputum of patients with bronchiectasis and healthy controls (*n* = 3 per group). (H) Comparison of caspase-1 p20 protein expression levels among the stable group, AE group, and healthy control group (*n* = 3 per group). Groups were compared by using paired *t* tests. Correlations were analyzed by using Pearson’s correlation analysis. AE, acute exacerbation; ROC, receiver operating characteristic; NEU%, neutrophil percentage.

Inflammasome activation is key to IL-1β release [12]. To determine the effects of NETs and PsA LPS on inflammasome activation, we first examined the sputum IL-1β levels at exacerbation. We enrolled 38 adults with bronchiectasis (median age: 54 years; 19 males) (Table 1 and Fig. 1). Sputum IL-1β levels were significantly greater at AE than in the stable state (median: 5,247.0 pg/ml vs. 2,356.1 pg/ml, *P* < 0.05; Fig. 2A), as were blood neutrophil percentages (71.9% vs. 64.6%, *P* < 0.05; Fig. 2B). In our cohort, 2 discrete AE episodes have been documented in 12 out of the 38 patients. Compared with stable state, sputum IL-1β levels were elevated during 2 discrete AE episodes (both *P* < 0.05; Fig. 2C). When stable-state and AE samples were combined, sputum IL-1β levels were correlated modestly with blood neutrophil percentages (*R*^2^ = 0.204, *P* < 0.05; Fig. 2D). We compared the diagnostic performance of IL-1β with serum C-reactive protein (CRP), which was available at 56 visits (32 in stable state and 24 in AE visits). Sputum IL-1β levels correlated modestly with CRP levels (*R*^2^ = 0.565, *P* < 0.001; Fig. 2E). Furthermore, baseline IL-1β levels could help distinguish AE from stable state (area under the curve [AUC]: 0.790, 95% CI: 0.688 to 0.891; Fig. 2F), while the AUC was 0.747 for blood neutrophil percentages (95% CI: 0.632 to 0.862).

**Table 1.** Inflammatory responses at paired stable state and exacerbation states in 38 patients with bronchiectasis. Boldface denotes *P* < 0.05.

Variables	Disease status ^a^		*P* value
	Stable	AE	
No. of patients	38	38	–
Age (years), median (IQR)	54 (38, 64)	–	–
Male sex, *n* (%)	19 (50%)	–	–
Body-mass index (kg/m^2^), mean (SD)	20.3 (3.0)	–	–
Ex- or current smokers, *n* (%)	5 (13%)	–	–
Etiology	–	–	–
Idiopathic, *n* (%)	14 (37%)	–	–
Post-infective, *n* (%)	16 (40%)	–	–
Primary ciliary dyskinesia, *n* (%)	4 (11%)	–	–
Asthma, *n* (%)	1 (3%)	–	–
Immunodeficiency, *n* (%)	2 (5%)	–	–
GERD-aspiration, *n* (%)	1 (3%)	–	–
Duration of symptoms (years), median (IQR)	19.3 (12.6, 29.2)	–	–
Exacerbation frequency in the previous year, median (IQR)	2.0 (1.8, 3.0)	–	–
FEV_1_ (%), mean (SD)	59.3 (18.1)	–	–
Bronchiectasis Severity Index, median (IQR)	10.0 (6.0, 13.0)	–	–
Medications	–	–	–
Mucolytics, *n* (%)	31 (82%)	–	–
Bronchodilators, *n* (%)	17 (45%)	–	–
Inhaled corticosteroids, *n* (%)	8 (21%)	–	–
Low-dose maintenance macrolides, *n* (%)	3 (8%)	–	–
Repeated detection of *Pseudomonas aeruginosa*, *n* (%)	15 (40%)	–	–
Repeated detection of PPMs, *n* (%)	6 (16%)	–	–
Detection of *Pseudomonas aeruginosa*, *n* (%)	18 (47%)	14 (37%)	0.486
Any positive sputum culture, *n* (%)	24 (63%)	25 (66%)	>0.999
Blood neutrophils (×10^9^ cells/l), mean (SD)	4.7 (1.8)	6.6 (3.3)	**<0.001**
Blood neutrophil (%), mean (SD)	64.6 (9.1)	71.9 (9.1)	**<0.001**
Sputum IL-1β (pg/ml), mean (SD)	2,356.1 (2,386.4)	5,247.0 (4,103.4)	**<0.001**

AE, acute exacerbation; IL-1β, interleukin (IL)-1β; PPMs, potentially pathogenic microorganisms; FEV_1,_ forced expiratory volume in 1 s; IQR, interquartile range; GERD, gastroesophageal reflux disease; SD, standard deviation

^a^ Each patient in the cohort provided a stable-state sample and a paired AE sample.

To assess NLRP3 inflammasome activation, we compared sputum caspase-1 p20 expression between bronchiectasis patients and healthy controls. Caspase-1 p20 levels were significantly higher at both AE and stable state compared with those in healthy controls (both *P* < 0.05; Fig. 2G and H). Overall, increased sputum IL-1β expression levels could differentiate AE from stable state and reflect blood neutrophilia in bronchiectasis.

### Inflammasome activation in blood neutrophils and NET production

To investigate the mechanisms underlying neutrophil activation, IL-1β release, and NET formation, we further enrolled 86 patients (4 were excluded because of systemic corticosteroid use, autoimmune disease or malignancy, Fig. 1). The AE and stable-state cohorts were balanced in terms of age, sex, smoking history, concomitant respiratory disease, and PsA colonization (Table 2). The etiology or PsA colonization status did not influence the results between the AE and stable-state cohorts. The leukocyte, neutrophil, and monocyte counts and CRP levels were significantly greater at AE than at stable state (all *P* < 0.05).

**Table 2.** Demographic and clinical characteristics of the 82 patients with bronchiectasis. Boldface denotes *P* < 0.05.

Variables	Patients with bronchiectasis		Total cohort	*P* value
	Stable ^a^	AE ^a^		
No. of patients	58	24	82	
Age (years), median (IQR)	49.7 (41.3, 63.2)	41.6 (34.6, 60.8)	48.8 (37.0, 62.4)	0.26
Male sex, *n* (%)	31 (53%)	10 (42%)	41 (50%)	0.94
Body mass index (kg/m^2^), mean (SD)	21.5 (3.5)	19.7 (1.8)	21.0 (3.2)	**0.004**
Ex- or current smokers, *n* (%)	5 (9%)	1 (4%)	6 (7%)	0.67
Duration of diagnosis (years), median (IQR)	14.0 (8.0, 23.8)	11.5 (10.0, 21.5)	13.5 (8.5, 23.0)	0.91
Etiology	–	–	–	0.80
Idiopathic, *n* (%)	40 (69%)	19 (79%)	59 (72%)	–
Post-infective, *n* (%)	10 (17%)	5 (21%)	15 (18%)	–
Primary ciliary dyskinesia, *n* (%)	1 (2%)	0 (0%)	1 (1%)	–
Asthma, *n* (%)	1 (2%)	0 (0%)	1 (1%)	–
Immunodeficiency, *n* (%)	4 (7%)	0 (0%)	4 (5%)	–
GERD-aspiration, *n* (%)	2 (3%)	0 (0%)	2 (2%)	–
Comorbidity	–	–	–	–
Allergic rhinitis, *n* (%)	5 (7%)	0 (0%)	5 (6%)	0.32
Sinusitis, *n* (%)	21 (36%)	12 (50%)	33 (40%)	0.32
Asthma, *n* (%)	12 (21%)	2 (8%)	14 (17%)	0.22
COPD, *n* (%)	5 (9%)	2 (8%)	7 (9%)	1.00
Bronchiectasis severity index, median (IQR)	4.5 (2.0, 8.0)	5.5 (2.0, 10.0)	4.5 (2.0, 8.0)	0.32
Mild, *n* (%)	2 (50%)	12 (50%)	41 (50%)	–
Moderate, *n* (%)	19 (33%)	3 (13%)	22 (26.8%)	–
Severe, *n* (%)	10 (17%)	9 (38%)	19 (23%)	–
FEV_1_/FVC (%), mean (SD)	66.5 (11.8)	70.9 (12.8)	67.8 (12.2)	0.15
FEV_1_% predicted (%), mean (SD)	82.5 (2.5)	81.7 (5.2)	82.3 (3.5)	0.46
≥80%, *n* (%)	19 (33%)	5 (21%)	24 (29%)	–
50–80%, *n* (%)	26 (45%)	13 (54%)	39 (48%)	–
<50%, *n* (%)	13 (22%)	6 (25%)	19 (23%)	–
Reiff scores, median (IQR) ^b^	7 (4, 11)	8 (6, 12)	8 (5, 12)	0.47
No. of exacerbations in the last year	28 (48%)	18 (75%)	46 (56%)	**0.027**
No. of hospital admissions in the last year	8 (14%)	7 (29%)	15 (18%)	0.10
Detection of *Pseudomonas aeruginosa*, *n* (%)	23.0 (40%)	7.0 (29%)	30.0 (37%)	0.45
Any positive sputum culture, *n* (%)	31.0 (54%)	12.0 (50%)	43.0 (52%)	0.81
*Pseudomonas aeruginosa* colonization (%)	14 (24%)	6 (25%)	20 (24%)	0.93
Blood cell counts (×10^9^ cells/l), mean (SD) ^b^	–	–	–	–
White blood cells	7.1 (2.2)	9.4 (3.2)	7.7 (2.7)	**0.004**
Neutrophils	4.4 (2.0)	6.5 (2.7)	5.1 (2.4)	**0.002**
Lymphocytes	2.0 (1.8)	1.9 (0.6)	1.9 (1.8)	0.60
Eosinophils	0.1 (0.1)	0.1 (0.1)	0.1 (0.1)	0.47
Monocytes	0.5 (0.2)	0.8 (0.4)	0.6 (0.3)	**0.006**
Sputum cell percentage (%), mean (SD) ^b^	–	–	–	**–**
Neutrophils	92.4 (11.3)	96.9 (0.3)	93.4 (10.1)	**0.036**
Lymphocytes	1.1 (0.9)	1.2 (0.6)	1.1 (0.8)	0.12
Eosinophils	3.9 (10.1)	0.9 (0.6)	3.3 (8.9)	0.20
Monocytes	2.6 (4.9)	1.0 (0.7)	2.2 (4.3)	0.10
C-reactive protein (mg/dl), mean (SD)	0.5 (0.6)	4.5 (4.5)	1.8 (3.2)	**<0.001**
Medications	–	–	–	–
Mucolytics, *n* (%)	46 (79%)	21 (88%)	67 (82%)	0.54
Bronchodilators, *n* (%)	26 (45%)	11 (46%)	37 (45%)	1.00
Inhaled corticosteroids, *n* (%)	6 (10%)	2 (8%)	8 (10%)	1.00
Low-dose maintenance macrolides, *n* (%)	6 (10%)	1 (4%)	7 (9%)	0.44

AE, acute exacerbation; IQR, interquartile range; SD, standard deviation; FEV_1,_ forced expiratory volume in 1 s; FVC, forced vital capacity; GERD, gastroesophageal reflux disease; COPD, chronic obstructive pulmonary disease

^a^ The stable cohort and the AE cohort are 2 separate cohorts.

^b^ Among patients in the stable-state cohort, 1 had unavailable Reiff scores, 3 had unavailable blood cell counts, and 27 had unavailable sputum cell percentages. Among the patients in the AE state, 1 had unavailable blood cell counts, and 15 had available sputum cell percentages.

We next quantified the expression of CD15, CD16, and CD66b, 3 core surface markers that modulate neutrophil maturation and activation, via flow cytometry. Compared with mild-to-moderate bronchiectasis, severe bronchiectasis significantly up-regulated CD66b and CD16 expression and blunted CD15 expression in blood neutrophils (all *P* < 0.05; Fig. S1A). Among patients with severe bronchiectasis, neutrophil CD66b expression was significantly increased, whereas CD15 expression was markedly depleted, at AE compared with stable state (both *P* < 0.05; Fig. S1B and C).

**Fig. 3.**
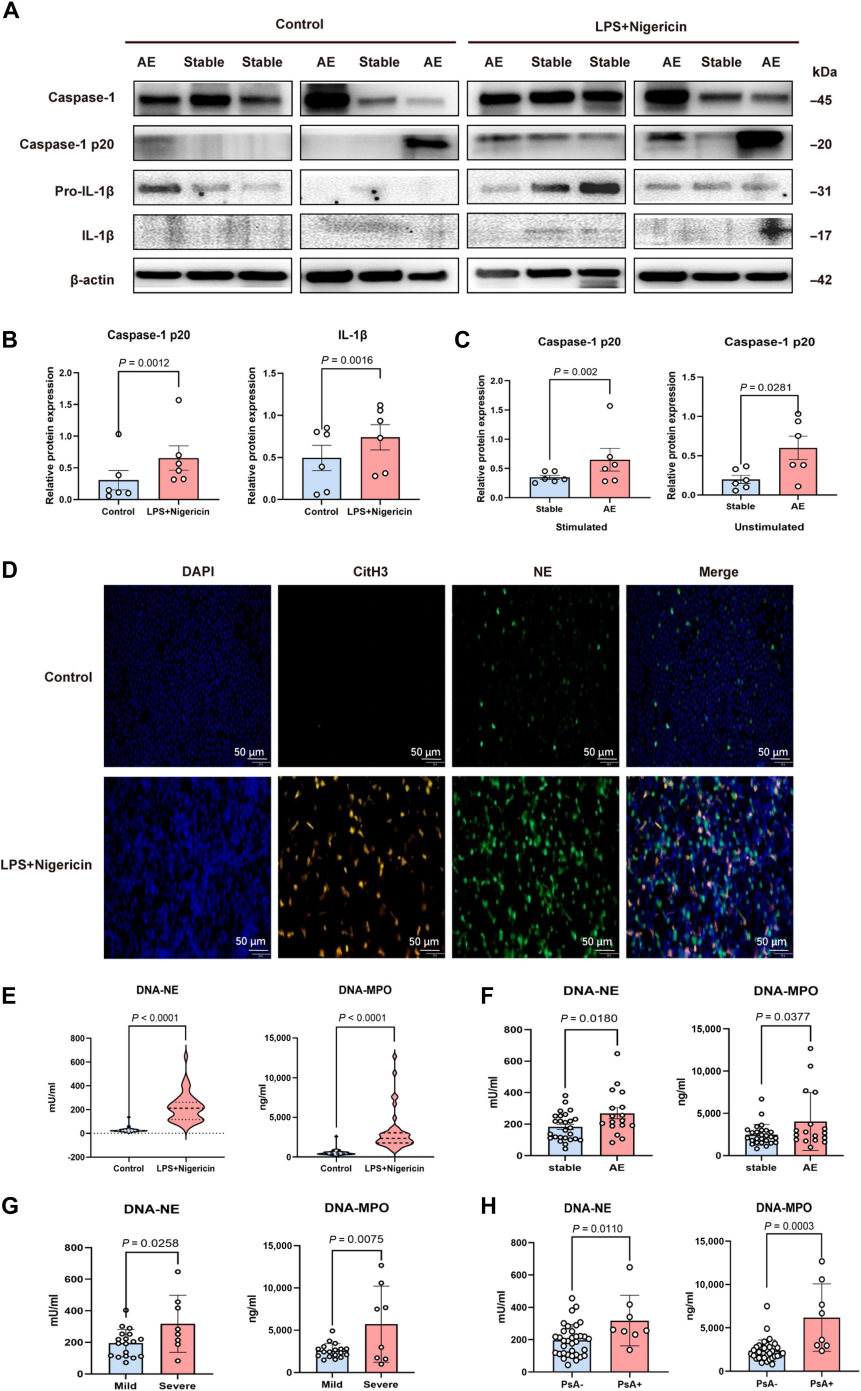
The activation of neutrophilic inflammasomes and NET formation in peripheral blood of bronchiectasis patients. Neutrophils from the peripheral blood of patients with bronchiectasis were stimulated with LPS (*PAO1*, 500 ng/ml) for 3 h, followed by nigericin (10 μM) stimulation for an additional hour. (A) Western blot analyses of caspase-1, caspase-1 p20, pro-IL-1β, IL-1β and endogenous control β-actin in blood neutrophils from patients with bronchiectasis (3 representative samples each at stable state and AE). (B) Comparison of caspase-1 p20 and IL-1β protein expression levels between the control group and the LPS + nigericin group (*n* = 6 per group). Groups were compared using independent *t* tests. (C) Comparison of caspase-1 p20 protein expression levels between the stable group and the AE group with or without stimulation. (*n* = 6 per group). Groups were compared using independent *t* tests. (D) Immunofluorescence staining of CitH3 (red), NE (green) and 4′,6-diamidino-2-phenylindole (DAPI, blue) expression in neutrophils from patients with bronchiectasis (400×, scale bar = 50 μm). (E) Comparison of DNA-NE and DNA-MPO concentrations between the control group and the LPS + nigericin group (*n* = 43 per group). (F) Comparison of DNA-NE and DNA-MPO concentrations between the stable group and the AE group (stable group: *n* = 26; AE group: *n* = 17). Groups were compared using independent *t* tests. (G) Comparison of DNA-NE and DNA-MPO concentrations between the mild bronchiectasis group and the severe bronchiectasis group in the stable state (mild group: *n* = 18; severe group: *n* = 8). Groups were compared using independent *t* tests. (H) Comparison of DNA-NE and DNA-MPO concentrations between the PsA-colonized group and noncolonized group (noncolonized group: *n* = 34; PsA-colonized group: *n* = 8). Groups were compared using independent *t* tests. AE, acute exacerbation; NETs, neutrophil extracellular traps; LPS, lipopolysaccharide; CitH3, citrullinated histone H3; NE, neutrophil elastase; MPO, myeloperoxidase; PsA, *Pseudomonas aeruginosa*.

To understand neutrophil inflammasome activation, we randomly selected and isolated blood neutrophils from 12 bronchiectasis patients (6 each at stable state and AE). Following stimulation with LPS (500 ng/ml), nigericin (10 μM) was added for stimulation. Both caspase-1 p20 and IL-1β levels were significantly increased following LPS and nigericin stimulation (all *P* < 0.05; Fig. 3A and B). Furthermore, inflammasome activation was more pronounced at AE than at stable state (*P* < 0.05; Fig. 3C). At AE, heightened activation of neutrophil inflammasomes was detected even without LPS stimulation. Specifically, neutrophil caspase-1 p20 levels were significantly elevated at AE than at stable state under unstimulated conditions (*P* < 0.05; Fig. 3C). NLRP3, ASC, and the active caspase-1 fragment (p20) were significantly higher in patients with bronchiectasis than in healthy controls (*P* < 0.05; Fig. S2A and B). Consistently, immunofluorescence staining confirmed increased NLRP3 and ASC expression in neutrophils during AEs compared with neutrophils from healthy controls (both *P* < 0.05; Fig. S3A to D), indicating up-regulation of key inflammasome components in bronchiectasis. There was a significant increase in the protein levels of NLRP3 and ASC in neutrophils from patients during AE compared with those isolated from the stable-state (both *P* < 0.05; Fig. S4 and B). Therefore, neutrophils isolated at AE exhibited persistent inflammasome activation, regardless of exposure to external stimuli.

Bronchiectasis patients exhibited increased formation of NETs (containing NE and citrullinated histone H3 [CitH3]) following LPS + nigericin costimulation, in contrast with the sporadic NE formation observed in unstimulated controls (Fig. 3D). Compared with the controls, bronchiectasis patients presented increased levels of DNA-MPO and DNA-NE (the cardinal NET protein complexes) (both *P* < 0.05; Fig. 3E). Following stimulation with LPS and nigericin, immunofluorescence analysis revealed that NETs could be induced in both healthy controls and patients with bronchiectasis. Notably, the quantity of NETs induced in bronchiectasis patients during AE was significantly larger than in healthy individuals (*P* < 0.05, Fig. S5A and B). Compared with those in the stable state, the DNA-NE and DNA-MPO levels were significantly greater at AE (both *P* < 0.05; Fig. 3F). Patients with severe bronchiectasis also exhibited notably higher levels of DNA-NE and DNA-MPO than did those with mild bronchiectasis at stable state (both *P* < 0.05; Fig. 3G). Therefore, NET formation is correlated with more severe bronchiectasis and AE onset. Both DNA-NE and DNA-MPO levels were significantly higher in patients with PsA colonization compared with those without (both *P* < 0.05; Fig. 3H), indicating that PsA drives NET elevation in patients with bronchiectasis.

### LPS induced inflammasome precursor marker activation and neutrophil chemokine production in bronchial epithelial cells

**Fig. 4.**
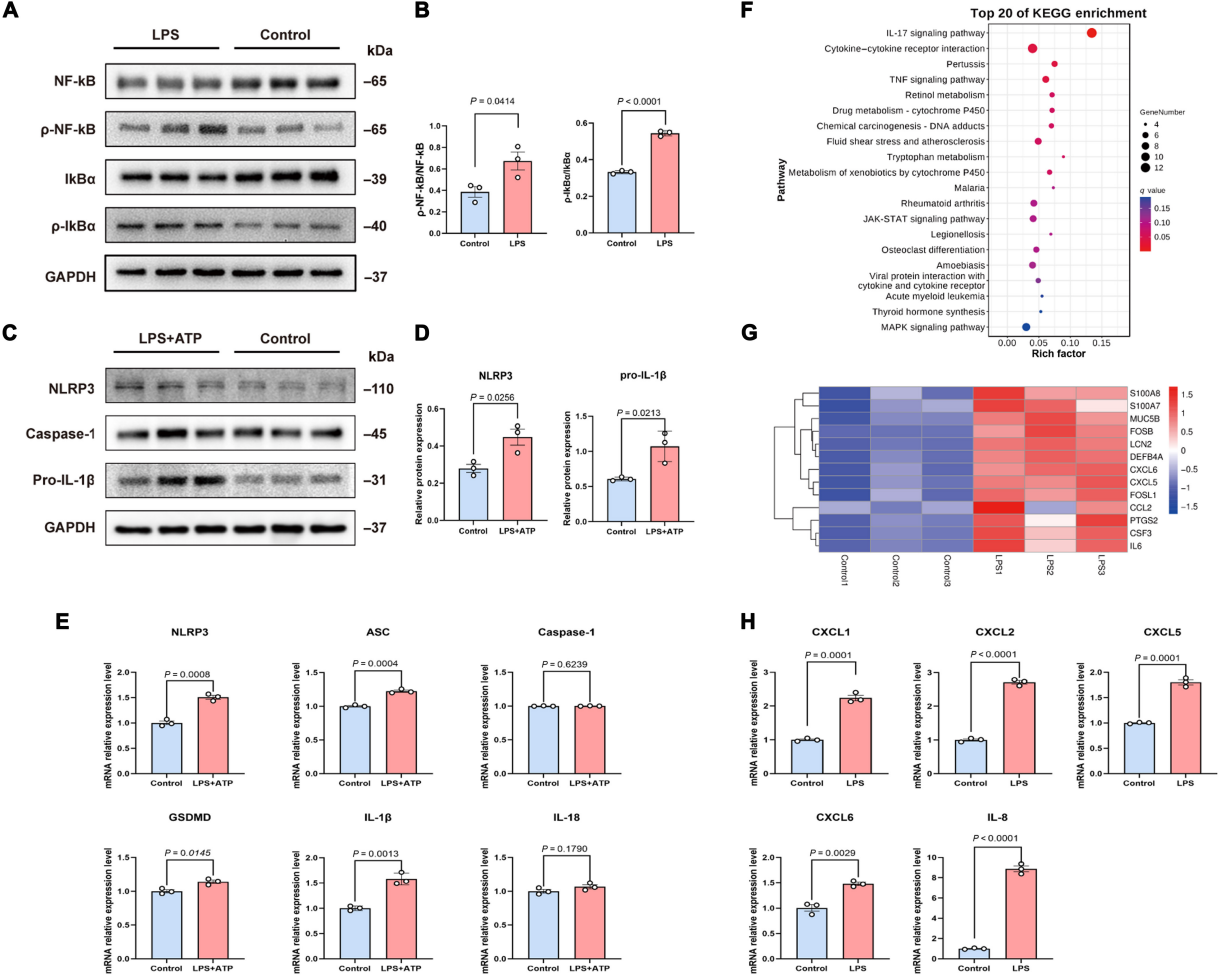
LPS-induced activation of the NLRP3 inflammasome, NF-κB pathway and neutrophilic chemokine pathway in airway epithelial cells of bronchiectasis patients. Differentiated primary human airway cells at the air–liquid interface were stimulated with LPS (*PAO1*, 20 μg/ml) for 24 h, followed by an additional hour of stimulation with ATP (5 mM). (A) Western blot analyses of NF-κB, ρ-NF-κB, IkBa, ρ-IkBa, and the endogenous control GAPDH in bronchial epithelial cells (*n* = 3 per group). (B) Comparison of the ρ-NF-κB/NF-κB and ρ-IkBa/IkBa ratios between the control group and the LPS group (*n* = 3 per group). (C) Western blot analyses of NLRP3, caspase-1, pro-IL-1β, and the endogenous control GAPDH in bronchial epithelial cells (*n* = 3 per group). (D) Comparison of the protein expression levels of NLRP3 and pro-IL-1β between the control group and the LPS + ATP group (*n* = 3 per group). (E) Comparison of NLRP3 inflammasome marker mRNA levels between the control and LPS + ATP groups (*n* = 3 per group). (F) KEGG pathway analysis showing differentially expressed genes that were identified between the control and LPS groups (top 20 genes for KEGG enrichment). (G) Heatmap of the differentially expressed genes in the cytokine–cytokine receptor interaction signaling pathway between the control and LPS groups. (H) Comparison of neutrophilic chemokine mRNA levels between the control group and the LPS group (*n* = 3 per group). Groups were compared by an unpaired *t* test. ATP, adenosine triphosphate; LPS, lipopolysaccharide; NLRP3, nucleotide-binding oligomerization domain-like receptor protein 3.

After 28 to 30 days of culture, epithelial cells derived from bronchiectasis patients developed a mature, pseudostratified, ciliated, columnar epithelium at the air–liquid interface (ALI). After 24 h of stimulation with PsA LPS, the NLRP3, IL-1β, IL-1α, IL-6, and tumor necrosis factor-α mRNA levels were significantly increased compared with those in the control group (all *P* < 0.05; Fig. S6), whereas the apoptosis-associated speck-like protein (ASC), caspase-1, GSDMD, and IL-18 mRNA levels did not significantly change. Additionally, the relative phosphorylation ratios of nuclear factor kappa-B (NF-κB) and the inhibitor of kappa B alpha (IκBα) in the LPS-stimulated group were notably greater than those in the control group (all *P* < 0.05; Fig. 4A and B), suggesting that NF-κB signaling pathway activation is the first signal involved in priming inflammasome activation.

We costimulated epithelial cells with adenosine triphosphate (ATP, 5 mM) for an additional hour. Compared with those in the control group, NLRP3 and pro-IL-1β protein expression levels were notably greater following LPS + ATP costimulation (all *P* < 0.05; Fig. 4C and D). However, based on caspase-1 p20 and IL-1β levels, inflammasome activation in epithelial cells was not observed following LPS + ATP costimulation. The NLRP3, ASC, GSDMD, and IL-1β mRNA levels were significantly greater than those in the controls (all *P* < 0.05; Fig. 4E), whereas the caspase-1 and IL-18 mRNA levels were comparable. Overall, inflammasomes within epithelial cells from bronchiectasis patients were incompletely activated despite LPS + ATP costimulation. We have also attempted to obtain airway epithelial cells from healthy individuals. The results show that in airway epithelial cells from healthy individuals, LPS stimulation led to an upward trend in the protein expression levels of inflammasome-related markers NLRP3, caspase-1, and pro-IL-1β compared with the blank control group; however, these differences were not statistically significant (*P >* 0.05, Fig. S7A and B).

We next conducted transcriptomic sequencing to profile global gene expression in epithelial cells with and without LPS stimulation (Fig. S8). Notably, gene expression levels within the cytokine–cytokine receptor interaction pathway differed substantially (*P* < 0.05, Fig. 4F). S100 calcium binding protein A8 (S100A8), S100A7, chemokine CXC ligand 5 (CXCL5), CXCL6, and granulocyte colony-stimulating factor were significantly up-regulated in the LPS-stimulated group (Fig. 4G). Additionally, quantitative polymerase chain reaction (qPCR) demonstrated that the CXCL1, CXCL2, CXCL6, and CXCL8 mRNA levels were increased in the LPS-stimulated group (all *P* < 0.05; Fig. 4H).

In summary, LPS induces chemokine release to facilitate neutrophil recruitment to the airway epithelium. Upon neutrophil activation, NETs might act as secondary signals to trigger inflammasome activity.

### LPS + NET costimulation induced tissue damage, mucus hypersecretion, and epithelial inflammasome activation

To confirm the abovementioned hypothesis, we treated differentiated epithelial cells from bronchiectasis patients with NETs, LPS, or LPS + NETs for 24 h.

**Fig. 5.**
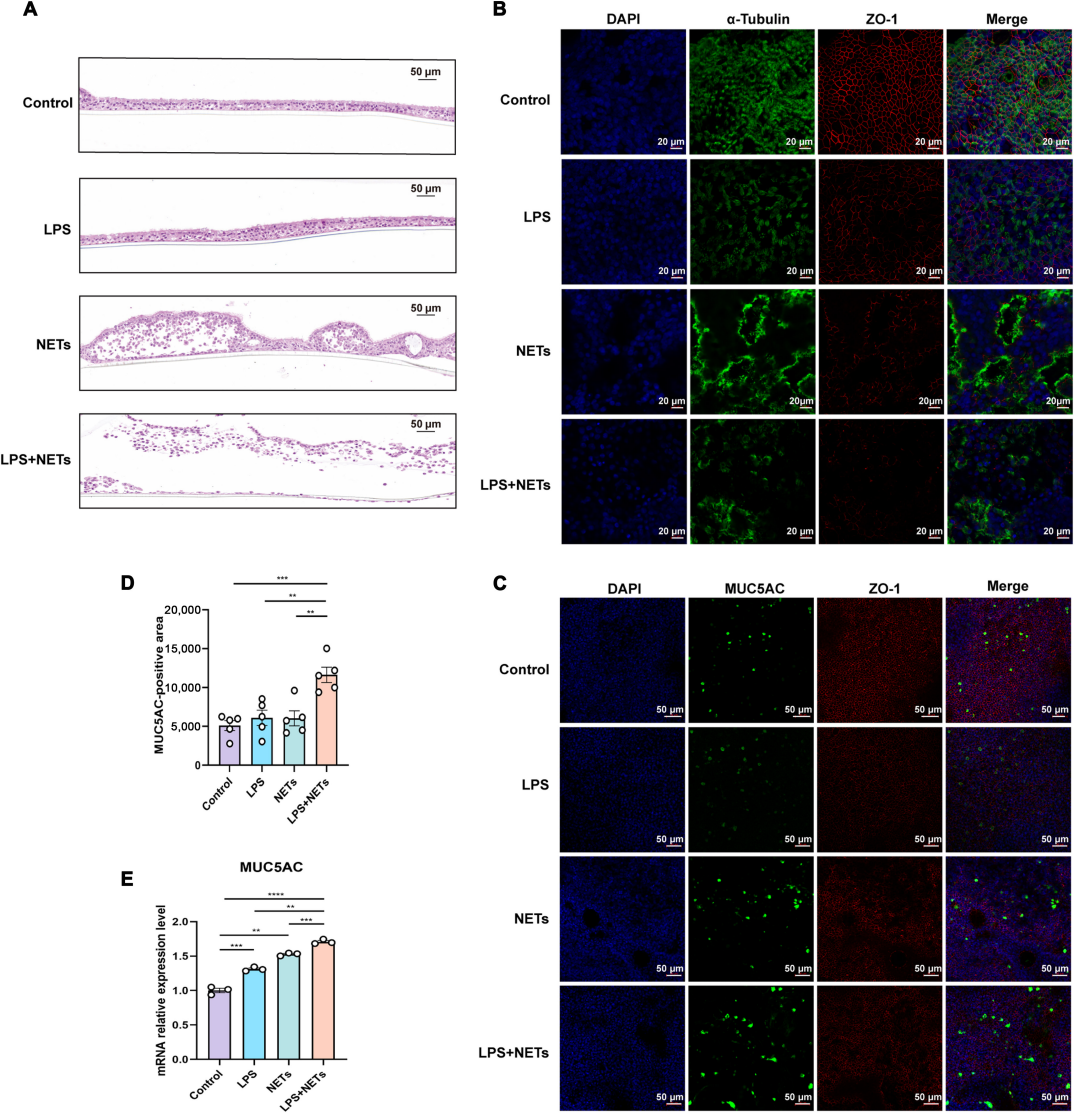
LPS and NETs induced tissue damage and mucus hypersecretion in airway epithelial cells of bronchiectasis patients. Differentiated primary human airway cells at the air–liquid interface were stimulated with LPS (*PAO1*, 20 μg/ml) and NETs (dsDNA = 500 ng/ml) for 24 h. (A) Representative images of the airway epithelium stained with H&E (200×, scale bar = 50 μm). (B) Immunofluorescence staining of tubulin (green), ZO-1 (red), and DAPI (blue) in airway epithelial cells (400×, scale bar = 20 μm). (C) Immunofluorescence staining of MUC5AC (green), ZO-1 (red), and DAPI (blue) in airway epithelial cells (200×, scale bar = 50 μm). (D) Semiquantitative analysis of MUC5AC-positive areas in the airway epithelium (data were obtained from 5 randomly selected fields per group). (E) Comparison of MUC5AC mRNA levels in bronchial epithelial cells among the different groups (*n* = 3 per group). Groups were compared by an unpaired *t* test. *P < 0.05; **P < 0.005; ***P < 0.0005; ****P < 0.0001. ZO-1, zona occludens-1; LPS, lipopolysaccharide; NETs, neutrophil extracellular traps; HE, hematoxylin–eosin; MUC5AC, Mucin 5AC.

Hematoxylin–eosin staining revealed markedly fewer ciliated cells and basal cells following exposure to NETs alone. LPS + NET costimulation aggravated epithelial damage, eliciting partial detachment of subepithelial cells (Fig. 5A). Immunofluorescence staining revealed a significant decrease in α-tubulin levels within ciliated cells and decreased zona occludens-1 expression (Fig. 5B). Furthermore, mucin 5AC (MUC5AC) secretion was increased upon NET or LPS stimulation alone, with a more prominent increase observed following LPS + NET costimulation (*P* < 0.05; Fig. 5C and D). Similarly, MUC5AC mRNA levels were significantly elevated in all stimulation groups (particularly the costimulation group) compared with the control group (*P* < 0.05; Fig. 5E).

**Fig. 6.**
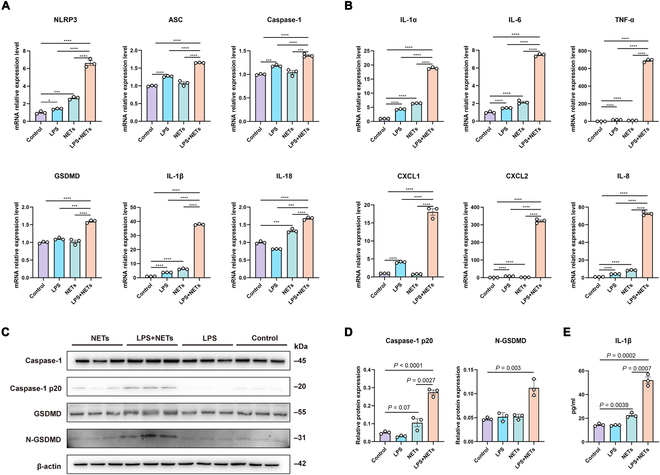
LPS and NETs trigger inflammasome activation and cytokine release in airway epithelial cells of bronchiectasis patients. (A) Comparison of NLRP3 inflammasome marker mRNA levels in bronchial epithelial cells among the different groups (*n* = 3 per group). (B) Comparison of the mRNA cytokine levels among the different groups (*n* = 3 per group). (C) Western blot analyses of caspase-1, caspase-1 p20, GSDMD, N-GSDMD, and the endogenous control β-actin in bronchial epithelial cells. (D) Comparison of caspase-1 p20 and N-GSDMD protein expression levels in bronchial epithelial cells among the different groups (*n* = 3 per group). (E) Comparison of IL-1β protein expression levels in bronchial epithelial cells among different groups (*n* = 3 per group). Groups were compared by an unpaired *t* test. *P < 0.05; **P < 0.005; ***P < 0.0005; ****P < 0.0001. NLRP3, nucleotide-binding oligomerization domain-like receptor protein 3; LPS, lipopolysaccharide; GSDMD, gasdermin D.

Furthermore, NET or LPS stimulation markedly up-regulated the mRNA expression of NLRP3 and IL-1β, but not other inflammatory biomarkers, compared with that in the control group (all *P* < 0.05; Fig. 6A). Compared with those in the control group, the levels of NLRP3, ASC, caspase-1, GSDMD, IL-1β, and IL-18 mRNAs were significantly increased in the costimulation group, with greater increases than those in the LPS-stimulated and NET-stimulated groups (both *P* < 0.05; Fig. 6B). Similarly, stimulation with NETs alone tended to up-regulate caspase-1 p20 expression (*P* = 0.07; Fig. 6C and D) and elicited a significant increase in IL-1β protein expression compared with that in the control group (*P* < 0.05; Fig. 6E). In contrast, compared with NETs alone, LPS + NET costimulation significantly increased caspase-1 p20, N-terminal gasdermin D (N-GSDMD), and IL-1β protein expression (all *P* < 0.05; Fig. 6D and E). Both inflammatory and neutrophil chemokine mRNA expression levels were significantly elevated in all stimulation groups compared with those in the control group, with the highest levels observed in the costimulation group (all *P* < 0.05; Fig. 6B).

Therefore, LPS + NET costimulation thoroughly activated epithelial inflammasomes, and NETs regulated inflammasome activation in bronchiectasis.

### Inflammasome-specific inhibitors ablated neutrophil inflammasome activation but not NET formation

Finally, we explored strategies for mitigating NET formation by ameliorating inflammasome activation or NET formation.

**Fig. 7.**
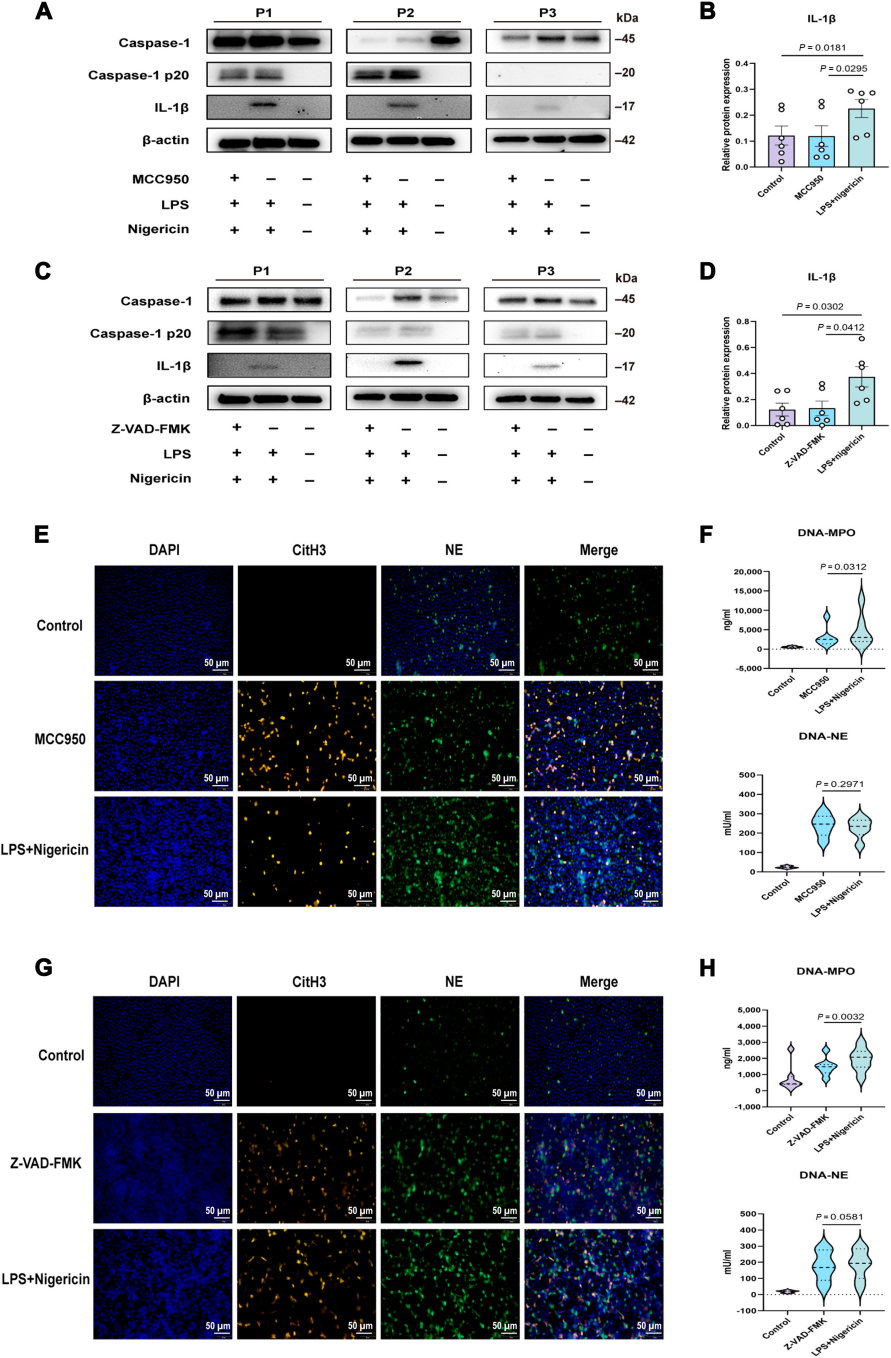
Effects of MCC950 and Z-VAD-FMK on inflammasome activation and NET formation in peripheral blood neutrophils of bronchiectasis patients. Neutrophils from the peripheral blood of patients with bronchiectasis were pretreated with the inhibitor MCC950 (10 μM) or Z-VAD-FMK (20 μM) for 1 h at 37 °C, followed by stimulation with LPS (*PAO1*, 500 ng/ml) for 3 h and with nigericin (10 μM) for an additional hour. (A and C) Western blot analyses of caspase-1, caspase-1 p20, IL-1β, and the endogenous control β-actin in neutrophils from different groups (*n* = 6 per group). (B and D) Comparison of caspase-1 p20 and IL-1β protein expression levels among the different groups (*n* = 6 per group). (E and G) Immunofluorescence staining of CitH3 (red), NE (green), and DAPI (blue) in neutrophils from patients with bronchiectasis (400×, scale bar = 50 μm) (*n* = 6 per group). (F and H) Comparison of DNA-NE and DNA-MPO concentrations among the different groups (*n* = 6 per group). Groups were compared by *t* tests and Mann–Whitney *U* tests according to their normal distribution. NETs, neutrophil extracellular traps; LPS, lipopolysaccharide; CitH3, citrullinated histone H3; NE, neutrophil elastase; MPO, myeloperoxidase.

We isolated neutrophils from bronchiectasis patients and treated them with MCC950 (an NLRP3 inhibitor, 10 μM) or Z-VAD-FMK (a caspase inhibitor, 20 μM), followed by stimulation with LPS and nigericin. Treatment with MCC950 or Z-VAD-FMK effectively suppressed mature IL-1β production (all *P* < 0.05; Fig. 7A and D). Neutrophils derived from bronchiectasis patients produced abundant NETs upon LPS or nigericin stimulation. Similar responses were observed in the groups treated with MCC950 and Z-VAD-FMK (Fig. 7E to G). There was a significant decrease in DNA-MPO (all *P* < 0.05; Fig. 7F to H), but not DNA-NE, levels following treatment with MCC950 or Z-VAD-FMK. Therefore, the inhibitory effects of MCC950 and Z-VAD-FMK on NET formation were limited.

**Fig. 8.**
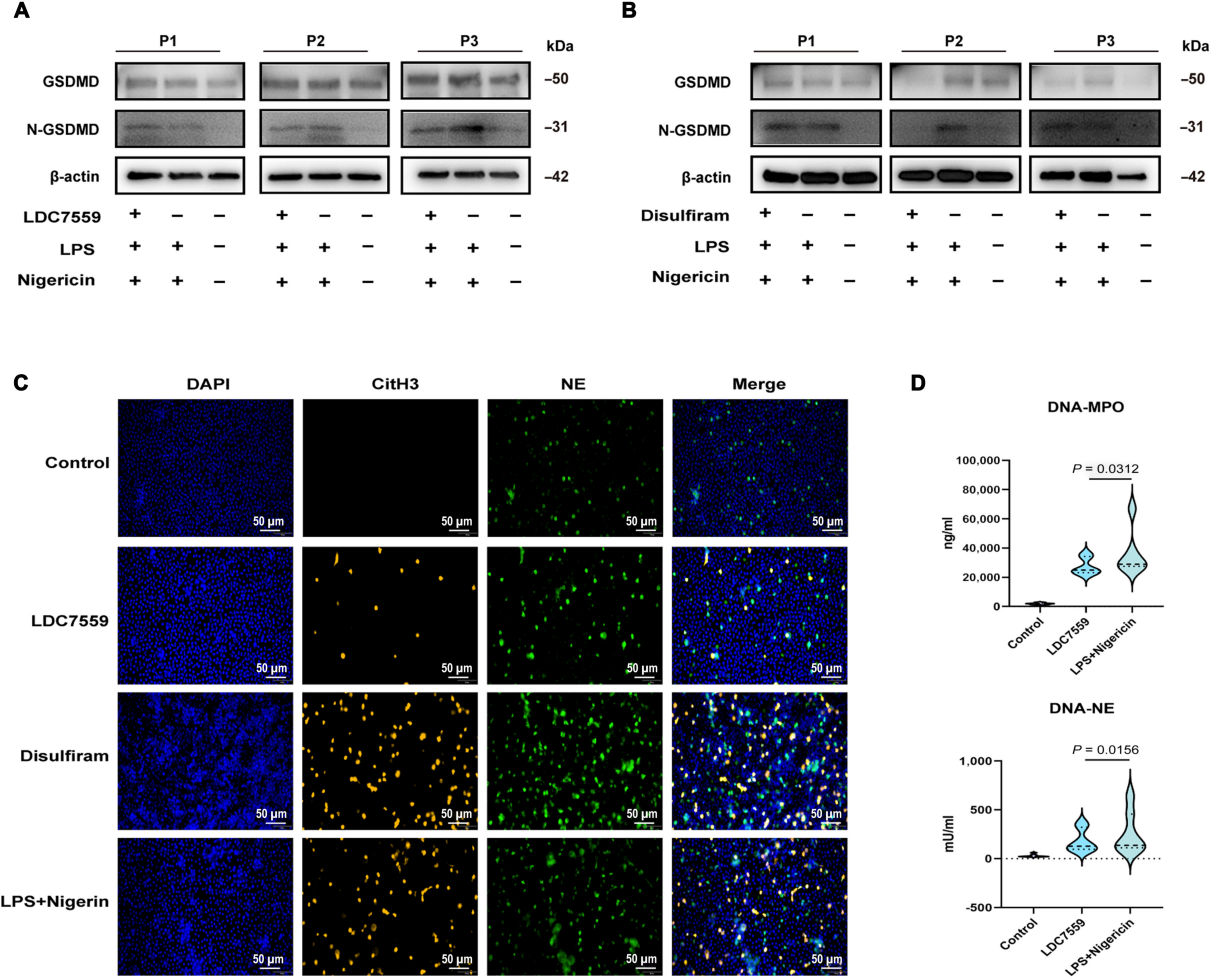
Effects of LDC7559 and disulfiram on inflammasome activation and NET formation in peripheral blood neutrophils of bronchiectasis patients. Neutrophils from the peripheral blood of patients with bronchiectasis were pretreated with the inhibitor LDC7559 (10 μM) or disulfiram (10 μM) for 1 h, followed by stimulation with LPS (*PAO1*, 500 ng/ml) for 3 h and with nigericin (10 μM) for an additional hour. (A and B) Western blot analyses of GSDMD, N-GSDMD, and the endogenous control β-actin in the neutrophils of patients with bronchiectasis (*n* = 6 per group). (C) Immunofluorescence staining of CitH3 (red), NE (green), and DAPI (blue) in neutrophils from patients with bronchiectasis (400×, scale bar = 50 μm) (*n* = 6 per group). (D) Comparison of DNA-NE and DNA-MPO concentrations among the different groups (*n* = 6 per group). Groups were compared by *t* tests and Mann–Whitney *U* tests according to their normal distribution. NETs, neutrophil extracellular traps; LPS, lipopolysaccharide; CitH3, citrullinated histone H3; NE, neutrophil elastase; MPO, myeloperoxidase; GSDMD, gasdermin D.

We further explored the effects of GSDMD inhibitors. Disulfiram reportedly inhibits the pore-forming activity of GSDMD on cell membranes [30], and LDC7559 prevents NET formation by targeting GSDMD [16]. We treated neutrophils from bronchiectasis patients with LDC7559 (10 μM) or disulfiram (10 μM) prior to stimulation. Interestingly, neither LDC7559 nor disulfiram markedly inhibited N-GSDMD formation within neutrophils (Fig. 8A and B). Furthermore, LDC7559 markedly decreased CitH3 production, whereas disulfiram did not (Fig. 8C). There was a notable reduction in the DNA-NE and DNA-MPO levels with increasing LDC7559 concentration (all *P* < 0.05; Fig. 8D). Overall, neither LDC7559 nor disulfiram significantly suppressed GSDMD expression. LDC7559, but not disulfiram, markedly suppressed NET formation.

**Fig. 9.**
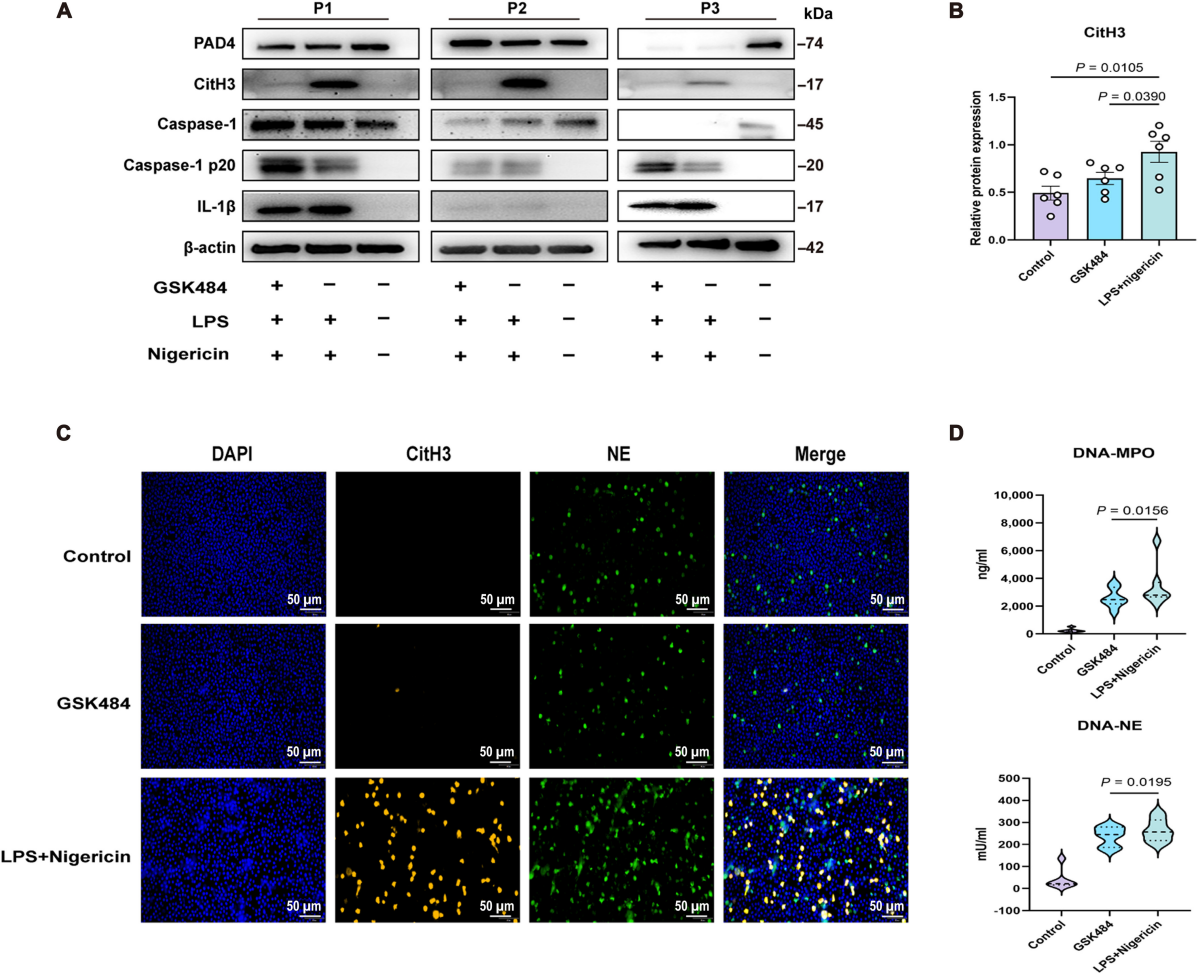
Effects of GSK484 on inflammasome activation and NET generation in peripheral blood neutrophils of bronchiectasis patients. Neutrophils from the peripheral blood of patients with bronchiectasis were pretreated with the inhibitor GSK484 (10 μM) for 1 h, followed by stimulation with LPS (*PAO1*, 500 ng/ml) for 3 h and with nigericin (10 μM) for an additional hour. (A) Western blot analyses of PAD4, CitH3, caspase-1, caspase-1 p20, IL-1β, and the endogenous control β-actin in the neutrophils of patients with bronchiectasis (*n* = 6 per group). (B) Comparison of CitH3 protein expression levels among different groups (*n* = 6 per group). (C) Immunofluorescence staining of CitH3 (red), NE (green), and DAPI (blue) in neutrophils from patients with bronchiectasis (400×, scale bar = 50 μm) (*n* = 6 per group). (D) Comparison of DNA-NE and DNA-MPO concentrations among the different groups (*n* = 6 per group). Groups were compared using *t* tests and Mann–Whitney *U* tests according to their normal distribution. NETs, neutrophil extracellular traps; LPS, lipopolysaccharide; CitH3, citrullinated histone H3; NE, neutrophil elastase; MPO, myeloperoxidase; PAD4, peptidylarginine deiminase type-4.

We ultimately blocked peptidyl arginine deiminase type IV (PAD4), an “upstream regulator” during NET formation, to inhibit chromatin decondensation and NET release [31]. We treated neutrophils with GSK484 (10 μM, a PAD4 inhibitor). In the GSK484-treated group, the CitH3 level was significantly reduced following LPS + nigericin costimulation (*P* < 0.05; Fig. 9A and B); however, GSK484 did not inhibit mature IL-1β formation. Immunofluorescence analysis indicated that GSK484 markedly decreased CitH3 generation (Fig. 9C). Additionally, enzyme-linked immunosorbent assays demonstrated a significant decrease in DNA-NE and DNA-MPO levels with GSK484 treatment (*P* < 0.05; Fig. 9D). Hence, PAD4 inhibitors effectively suppressed NET production but had no significant effect on inflammasome activation.

## Discussion

**Fig. 10.**
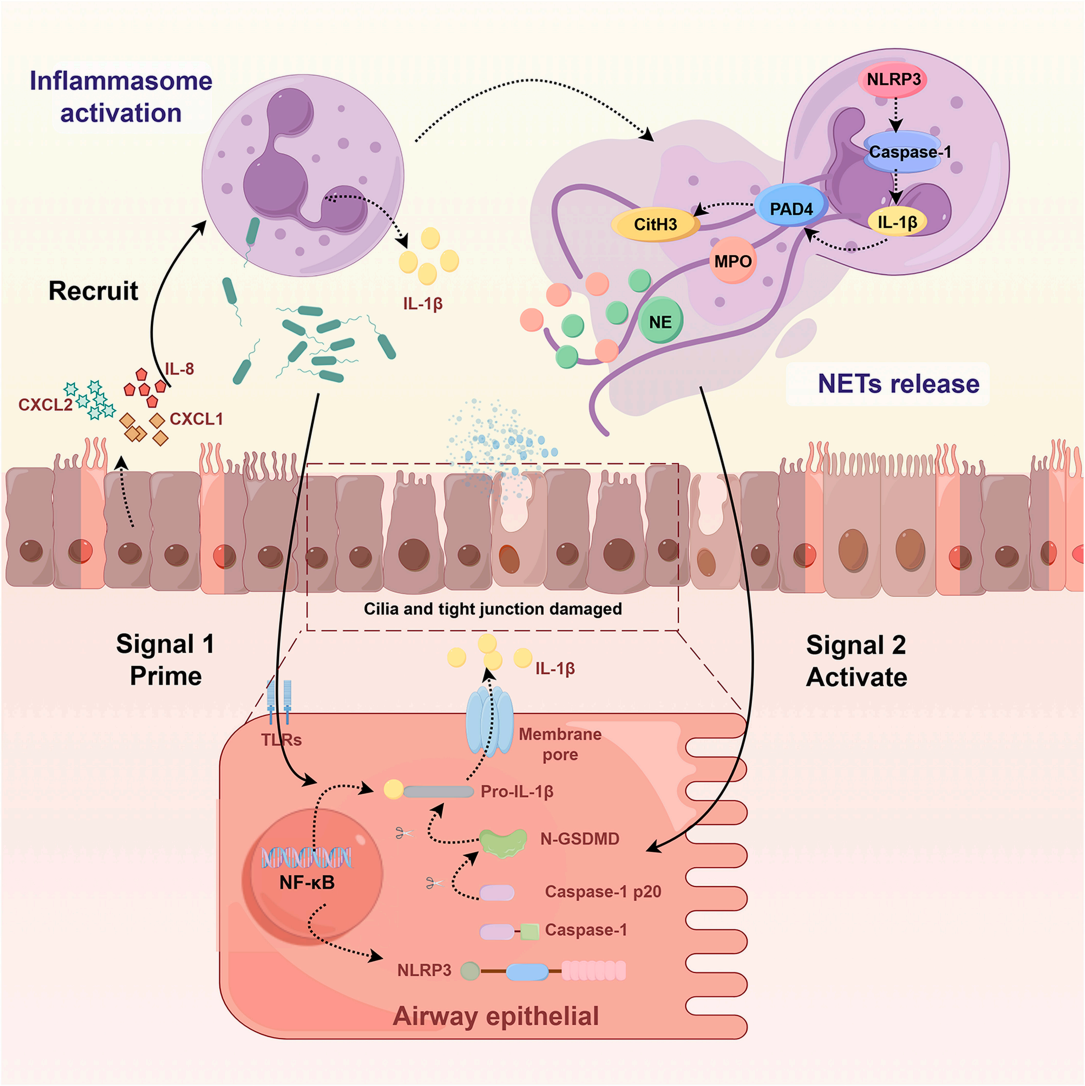
A schematic diagram of the mechanisms for inflammasome activation in bronchiectasis. Stimulation of airway epithelial cells in patients with bronchiectasis with *Pseudomonas aeruginosa* LPS initiates the activation of the NF-κB signaling pathway, resulting in elevated levels of NLRP3 and pro-IL-1β expression. This cascade of events leads to excessive chemokine release and neutrophil infiltration. Patients with bronchiectasis demonstrate prominent activation of neutrophil inflammasomes, resulting in massive NET formation following neutrophil activation. Furthermore, NETs could disrupt airway epithelial cell cilia and tight junctions, increase mucin MUC5AC levels, and provide a secondary signal for triggering inflammasome activation. In conjunction with LPS, this dual activation fully triggers the inflammasome in airway epithelial cells, exacerbating the inflammation and damage and chemokine release, thereby eliciting a “vicious cycle” in patients with bronchiectasis. NETs, neutrophil extracellular traps; LPS, lipopolysaccharide; CitH3, citrullinated histone H3; NE, neutrophil elastase; MPO, myeloperoxidase; PAD4, peptidylarginine deiminase type-4; GSDMD, gasdermin D; NLRP3, nucleotide-binding oligomerization domain-like receptor protein 3; MUC5AC, mucin 5AC.

Our study focused on inflammasome activation and NET formation in bronchiectasis. PsA LPS activated the NF-κB signaling pathway, resulting in the up-regulation of NLRP3 and pro-IL-1β and subsequent chemokine release and neutrophil infiltration. Additionally, PsA LPS induced inflammasome activation in neutrophils and triggered NET release. These NETs served as secondary signals, driving robust epithelial inflammasome activation, which exacerbated inflammation and airway damage (Fig. 10). Therapeutic interventions targeting multiple checkpoints could help mitigate excessive neutrophilic inflammation and tissue destruction in bronchiectasis.

Previous studies have revealed that neutrophil heterogeneity is affected by differentiation status and surface molecule expression. Compared with those from healthy individuals, blood neutrophils from bronchiectasis patients presented distinct traits, including prolonged survival, delayed apoptosis, increased CD62L shedding, and elevated CD11b expression [32]. In patients with severe bronchiectasis, blood neutrophils at AE exhibited higher CD66b and CD16 levels and reduced CD15 expression, indicating the release of activated neutrophil subpopulations. Additionally, bacteria reportedly activate neutrophil inflammasomes, leading to excessive IL-1β release [33–35]. Here, caspase-1 p20 and IL-1β protein levels in neutrophils were markedly elevated following LPS + nigericin costimulation, and AE was associated with more pronounced activation of inflammasomes than was the stable state. Notably, neutrophils at AE displayed heightened activation of the NLRP3 inflammasome in the absence of external stimuli. These findings echo the published literature, highlighting the sustained activation of inflammasomes in blood macrophages among patients with severe coronavirus disease 2019 [36]. Our study also revealed an intimate association between NLRP3 inflammasome activation and NET formation. LPS + nigericin costimulation resulted in more prominent NET formation by activated neutrophils from bronchiectasis patients. The higher levels of DNA-NE and DNA-MPO in patients with AE and severe bronchiectasis indicated that neutrophilic inflammation and NET formation are candidate biomarkers for evaluating bronchiectasis severity. In light of the small sample size and single-center, cross-sectional design, multicenter research needs to be conducted to validate and generalize the results. Longitudinal, within-subject monitoring of inflammasome activation markers alongside established inflammatory indicators such as CRP and procalcitonin from stable state through successive exacerbation and recovery phases will be critical to establish its kinetic profile and confirm its usefulness for predicting and monitoring AEs.

The lack of an ideal animal model for bronchiectasis has hindered the understanding of the pathophysiologic mechanisms involved. Here, we successfully isolated bronchial epithelial cells from bronchiectasis patients and generated mature pseudostratified ciliated epithelia at the ALI, followed by LPS + NET costimulation to replicate the airway milieu in bronchiectasis. Although costimulation activated the epithelial NF-κB signaling pathway, the expected increase in NLRP3 and Pro-IL-1β levels did not fully up-regulate IL-1β expression. Our observations are in line with previous reports. Direct stimulation with ATP and LPS failed to fully activate the NLRP3 inflammasome in both HBECs and the 16HBE cell line, while direct infection with PsA induced only minimal activation [37]. Despite the inflammasome activation in bronchial epithelial cell lines (NCI-H292 and 16HBE), pyroptosis is not evident in primary human bronchial epithelial cells under similar stimulation conditions [38]. Hence, a more robust and potent secondary signal is needed for complete epithelial inflammasome activation. In comparison, neither LPS nor the SARS-CoV-2 envelope protein could activate epithelial inflammasomes thoroughly. However, NETs acted as a secondary signal to increase inflammasome activation from blood mononuclear cells in patients with coronavirus disease 2019 [38]. Here, our transcriptomic analysis revealed a significant up-regulation of neutrophil chemotactic factors, with markedly increased mRNA expression levels of IL-8 (CXCL8), CXCL1, CXCL2, and CXCL6 compared with the control group. IL-8, also known as CXCL8, is secreted predominantly by epithelial cells, exerts strong chemotactic activity on neutrophils, and plays a central role in regulating the inflammatory responses [39]. The chemokine receptor CXCR2 engages multiple ligands—including CXCL1, CXCL2, CXCL3, CXCL5, CXCL6, CXCL7, and CXCL8—to promote neutrophil infiltration into the lungs [40]. This process facilitates the recruitment of circulating neutrophils into lung tissue and drives neutrophilic inflammation in the airway epithelium, suggesting that activated neutrophils may act as a secondary signal amplifying inflammasome activation within the bronchiectatic epithelium. NETs derived from adult-onset Still’s disease reportedly activate the NLRP3 inflammasome within macrophages [41,42]. NETs, which contain multiple enzymes including NE, can disrupt tight junctions [43], leading to increased permeability and decreased transepithelial electrical resistance (TEER) [44] and ciliary function, and facilitate mucin hypersecretion [45,46]. In our study, NETs resulted in excessive IL-1β secretion in bronchiectasis patients, and LPS + NET costimulation more prominently activated inflammasomes, indicating that NETs constitute the core secondary signal for inflammasome activation in bronchiectasis. Furthermore, LPS + NET costimulation aggravated epithelial cell disruption and promoted mucus hypersecretion. Therefore, costimulation with NETs and pathogens substantially exacerbated tissue damage and airway inflammation in patients with bronchiectasis.

We additionally assessed whether specific inflammasome inhibitors could abrogate neutrophil inflammasome activation and NET formation. Treatment with MCC950 and Z-VAD-FMK effectively attenuated neutrophil inflammasome activation in bronchiectasis. Nevertheless, NET formation was not entirely abrogated, with both inhibitors demonstrating inhibitory effects on DNA-MPO only. Research has indicated a notable decrease in NET formation in NLRP3 knockout mice under sterile conditions [17]. Furthermore, knockout of the caspase-11 gene significantly decreased NET formation in mice [15]. However, caspase inhibitors such as Z-VAD-FMK or VX-765 did not effectively attenuate NET formation in human and mouse neutrophils [16], which echoed our findings in bronchiectasis.

Disulfiram, a clinically approved agent for alcohol dependence, has been reported to inhibit GSDMD-mediated pore function [30], whereas the small-molecule compound LDC7559 was initially proposed to block GSDMD-dependent NET release [16]. In our study, treatment of patient-derived neutrophils with either disulfiram or LDC7559 did not markedly affect N-GSDMD production. Notably, LDC7559 reduced NET release, whereas disulfiram showed no discernible effect. Consistent with this, previous work has demonstrated that LDC7559 suppresses NETosis without preventing GSDMD cleavage or pyroptosis, instead identifying phosphofructokinase as its actual binding target [47]. Similarly, other studies have shown that while NLRP3 activation promotes GSDMD cleavage and IL-1β maturation, GSDMD is dispensable for PMA-induced NETosis [18], and GSDMD-deficient neutrophils retain NET-releasing capacity comparable to wild-type cells [19]. Our findings, together with prior evidence, suggest that activation of the NLRP3–caspase-1 axis in bronchiectasis neutrophils facilitates phenotypic activation and NET formation, but inflammasome signaling is not indispensable for NET release. Moreover, because the current GSDMD-targeting agents fail to effectively block GSDMD activation in patient-derived neutrophils, whether GSDMD constitutes a viable therapeutic target for controlling NETosis in bronchiectasis warrants further investigation.

PAD4 is a critical regulator of NET formation and has been described as a “master switch” that controls broad NET-associated protease activity. Increased PAD4 expression has been implicated in COPD, where elevated gene and protein expression levels in the lungs strongly correlate with the severity of pulmonary function decline and symptom burden [48]. In our study, the PAD4 inhibitor GSK484 suppressed NET formation but did not abolish mature IL-1β expression. This contrasts with the direct inhibition of inflammasome activation (upstream signaling), which failed to effectively block NET release, highlighting the greater efficacy of targeting PAD4 (downstream signaling). Clinically, single DNase therapy provides only limited benefits, and NE inhibitors act selectively on protease activity without addressing NET formation [29]. In support of our findings, PAD4 knockout significantly mitigated NET formation in a bleomycin-induced fibrosis mouse model [49], suggesting that PAD4 represents a promising therapeutic target for chronic neutrophilic airway diseases. Collectively, our data indicate that PAD4 inhibition may hold therapeutic potential in bronchiectasis.

Our study has certain limitations. First, it is a small-sample, single-center, cross-sectional study; larger, multi-center studies are needed to further validate these findings. Another major limitation is the lack of mature and suitable animal models for bronchiectasis; nonetheless, all samples were directly obtained from patients, thereby providing a reliable reflection of the true pathophysiological mechanisms of the human disease. In addition, the absence of pharmacologic or genetic inhibition of key components (e.g., NLRP3 and GSDMD) in epithelial cells during NET exposure has hindered our observation to fully confirm the causality. In future studies, we will develop suitable mouse models to validate the therapeutic potential of relevant targets. These strategies include the use of small-molecule inhibitors, biologics targeting upstream activators, and modulators of inflammasome assembly and signaling. We believe that targeting NLRP3 offers promising translational potential to alleviate chronic inflammation and tissue damage in bronchiectasis patients.

In summary, this study identifies a novel mechanism driving increased airway inflammation in bronchiectasis and calls for a comprehensive strategy to counteract both NLRP3 inflammasome activation and NET formation to manage neutrophilic inflammation, thereby providing new therapeutic targets for the pathogenesis of bronchiectasis.

## Materials and Methods

See the Supplementary Materials for detailed methods.

### Study population

Between August 2017 and December 2023, we enrolled 124 adults with bronchiectasis confirmed by high-resolution computed tomography at The First Affiliated Hospital of Guangzhou Medical University. After patients signed the informed consent form, sputum or blood samples were collected without the use of antibiotics.

The inclusion criteria, sample collection method, and definitions of AE and disease severity are provided in the Supplementary Materials.

### Study design

Based on a retrospective cohort (August 2017 to December 2021), we assessed the expression levels of mature IL-1β (17 kDa) in the sputum supernatant of 38 patients with bronchiectasis at stable state and AE by using multiplex immunoassay kits according to the manufacturer’s instructions (Bio-Rad, USA). We further recruited 86 patients in a cross-sectional cohort (April–December 2023) to collect blood or sputum samples at stable state or AE to evaluate NLRP3 inflammasome activation and NET formation (Fig. 1). Bronchiectasis etiology and bacteria colonization status were assessed at baseline. We defined repeated detection of the same pathogenic bacteria as the isolation of the same bacteria at least twice within a year, at intervals of 3 months or more.

### Neutrophil isolation and flow cytometry

Peripheral blood (20 ml) was subjected to Ficoll–Paque density gradient centrifugation (Cytiva, USA) to obtain the granulocyte layer. Erythrocytes were removed using red blood cell lysis buffer (BioLegend, USA), and neutrophils were purified using the EasySep Human Neutrophil Isolation Kit (STEMCELL Technologies, USA) and an LSR Fortessa (BD, USA). We adopted Flow Jo 10.6 (BD, USA) for analysis. Antibodies against CD45 (2D1-APC-Cy7), CD11b (ICRF44-FITC), CD15 (HI98- PE-CY7), CD16 (3G8-PerCP/Cyanine 5.5), CD62L (DREG-56-PE), and CD66b (G10F5- APC) (BD, USA) were used for neutrophil characterization.

### Assessment of the neutrophil inflammasome

An aliquot of protein was subjected to sodium dodecyl sulfate–polyacrylamide gel electrophoresis (SDS–PAGE), followed by transfer to a polyvinylidene fluoride (PVDF) membrane. After blocking with 5% skim milk, the membrane was incubated with primary antibodies against IL-1β (CST), cleaved-IL-1β (CST), GSDMD (CST), cleaved N-terminal GSDMD (Abcam), caspase-1 (Abcam), and β-actin (CST) overnight.

### Detection of NETs

Immunofluorescence staining with CitH3 (Abcam, USA, 1:500) and NE (Abcam, 1:200) was employed to assess NET formation. Quantitative NET assessment [50] was performed using DNA-NE and DNA-MPO. After removal of the neutrophil supernatant, NETs were degraded with S7 Nuclease Assay Reagent (15 U/ml; Cayman Chemical), followed by the addition of EDTA (0.5 mmol/l; Cayman Chemical) for quenching. A NETosis Assay Kit (Cayman Chemical) [51] was used for NET-associated NE, whereas an MPO ELISA Kit (Cusabio, Wuhan, China) was used for NET-associated MPO. The PAD4 (Abcam) and CitH3 (Abcam) proteins were quantified by Western blotting.

### NET purification

Neutrophils were stimulated with LPS (*P. aeruginosa* PAO1 strain, 500 ng/ml) for 3 h, followed by 1 h of stimulation with nigericin (10 μM) to induce NET formation. The culture medium was removed, and the NET layer was carefully scraped off and subjected to vigorous mixing. The sample was subsequently centrifuged at 450 × *g* for 10 min, after which the supernatant was collected. The dsDNA was quantified using the Quant-iT PicoGreen dsDNA Assay Kit.

### Inhibition assays

The inflammasome inhibitors Z-VAD-FMK (20 μM), MCC950 (10 μM), LDC7559 (10 μM), and disulfiram (10 μM) and the PAD4 inhibitor GSK484 (10 μM) were incubated for 1 h at 37 °C, followed by stimulation with LPS (*P. aeruginosa* PAO1 strain, 500 ng/ml) for 3 h and subsequent stimulation with nigericin (10 μM) for 1 h.

### ALI culture

We isolated bronchial epithelial cells via low-temperature enzymatic digestion from the bronchial tissues of bronchiectasis patients who underwent lobectomy, segmentectomy, or lung transplantation (Table S1). We seeded 4 × 10^4^ epithelial cells in Transwell inserts (Corning, 3470) and cultured them with PneumaCult-Ex Medium (STEMCELL Technologies, USA) to facilitate proliferation. Once the cells reached 100% confluence, they were transitioned to PneumaCult–ALI Medium (STEMCELL Technologies, USA) to induce differentiation for 28 to 30 days.

### Detection of the inflammasome

An aliquot of protein was subjected to SDS–PAGE, blotted onto PVDF membranes, and probed with specific antibodies against IκBα (CST), phospho-IκBα (CST), NF-κB p65 (CST), phospho-NF-κB p65 (CST), NLRP3 (CST), cleaved-IL-1β (CST), GSDMD (CST), cleaved N-terminal GSDMD (Abcam), caspase-1 (Abcam), β-actin (CST), and GAPDH (CST) via incubation overnight. qPCR was used to quantify the levels of the NLRP3, ASC, caspase-1, GSDMD, IL-1β, and IL-18 mRNAs.

### RNA sequencing and qPCR

Bronchial epithelial cells from bronchiectasis patients were stimulated with PsA-derived LPS (20 μg/ml) or left untreated. RNA was extracted, and a cDNA library was prepared using the Hieff NGS Ultima Dual-mode mRNA Library Prep Kit for Illumina (Yeasen Biotechnology [Shanghai] Co., Ltd.). Sequencing was conducted on the Illumina Nova 6000 platform (Gene Denovo Biotechnology). Neutrophilic chemokine mRNAs (CXCL1, CXCL2, CXCL5, CXCL6, and IL-8 mRNAs) were quantified by qPCR.

### Evaluation of epithelial damage

Paraffin sections of differentiated epithelial cells were prepared and stained with hematoxylin–eosin. The ALI cultures were subjected to immunofluorescence staining with primary antibodies against MUC5AC (Thermo Fisher Scientific), ZO-1 (CST), and acetylated tubulin (Abcam).

### Statistical analysis

The data were analyzed using SPSS 26 (SPSS Inc., Chicago, USA) and GraphPad Prism 10.0 (GraphPad Inc., San Diego, USA). Continuous variables were presented as mean (standard deviation [SD]) or median (interquartile range [IQR]), whereas counts and proportions were used for categorical variables. Between-group comparisons for continuous outcomes were made using *t* tests or nonparametric tests. Categorical variables are expressed as frequencies and percentages, with between-group differences evaluated by the chi-square test or Fisher’s exact test. Receiver operating characteristic curves were used to display the diagnostic value of sputum IL-1β and blood neutrophil percentages in distinguishing AE from stable state, along with the AUC and 95% confidence interval (95% CI). A *P* value of less than 0.05 was considered statistically significant.

## Ethical Approval

Ethics approval was obtained from the Ethics Committee of The First Affiliated Hospital of Guangzhou Medical University (Medical Ethics: [2012] No. 33; [2020] No. 156; ES-2023-001-01; [2024] No. 236; ES-2024-062-01). All participants provided written informed consent.

## Data Availability

Upon reasonable request, relevant information about the data will be provided directly by the corresponding author.
