# Supplementary material for: NLRP3 Inflammasome Activation Modulates Neutrophil Extracellular Trap Formation and Aggravates Airway Inflammation in Bronchiectasis
Source: Research (Wash D C). 2025 Oct 17;8:0958. doi: 10.34133/research.0958 (PMC12696696; doi:10.34133/research.0958)
Supplement: Supplementary 1 — Supplementary Methods Figs. S1 to S8 Table S1 [file research.0958.f1.zip › Supplementary Materials and Methods-20250923.docx]

**Supplementary Materials and Methods**

**NLRP3 Inflammasome Activation Modulates Neutrophil Extracellular Traps Formation and Aggravates Airway Inflammation in Bronchiectasis**

**Supplementary Methods**

***Recruitment and clinical assessment of study participants.***

Between August 2017 and December 2023, we enrolled 124 adult patients with bronchiectasis (at stable or acute exacerbation states) confirmed by the chest high-resolution CT (HRCT) scan imaging which was performed at the First Affiliated Hospital of Guangzhou Medical University. Clinically significant bronchiectasis was diagnosed as the presence of high-resolution computed tomography (HRCT) manifestations (an inner airway-artery diameter ratio of 1.5 or more, an outer airway-artery diameter ratio of 1.5 or more, a lack of tapering of the airways, and visibility of airways in the periphery) which were compatible with the respiratory symptoms (particularly daily cough, chronic mucopurulent or purulent sputum, a history of exacerbations)^[1]^. A stable state was defined as no clinical deterioration and no antibiotic use (except low-dose macrolides) for 4 weeks prior to enrollment. Acute exacerbation^[2]^ was characterized by a significant worsening of three or more symptoms for at least 48 hours, requiring a change in management.

After all patients signed the informed consent form, sputum or blood samples were collected without using antibiotics, and AE samples were collected on the first day of AE. We rated the radiologic severity of bronchiectasis using modified Reiff score^[3]^ and rated the bronchiectasis severity by using the Bronchiectasis Severity Index (BSI)^[4]^. Based on the BSI, patients were categorized into mild bronchiectasis (BSI ≤4), moderate bronchiectasis (4<BSI≤8) or severe bronchiectasis (BSI ≥9).

Exclusion criteria comprised: (1) An age of less than 18 years; (2) Active pulmonary tuberculosis; (3) Lung malignancy; (4) Cystic fibrosis and other hereditary pulmonary diseases; (5) Patients with autoimmune diseases or other immunodeficiencies (organ or bone marrow transplantation, immunosuppressant therapy, radiotherapy and chemotherapy within half a year, oral glucocorticoids within the past 4 weeks, splenectomy, etc.).

All participants signed written informed consent. Ethics approval was obtained from the Ethics Committee of The First Affiliated Hospital of Guangzhou Medical University (Medical Ethics [2012] the 33th; Medical Ethics [2020] the 156th).

***Study design***

Based on a retrospective observational cohort (August 2017 to December 2021), we assessed the expression levels of mature IL-1β (17kDa) in the sputum supernatant among 38 patients with bronchiectasis at both stable-state and acute exacerbation state. We further recruited 86 bronchiectasis patients in a cross-sectional cohort (April- December 2023) to collect blood or sputum samples during stable or acute exacerbation states to evaluate NLRP3 inflammasome activation and NETs formation.

Healthy subjects included the patients’ companions, medical staff, and those who underwent health check-up. The healthy subject should have normal chest X-ray and spirometry, and have no respiratory symptoms, severe systemic diseases, or antibiotic use within 4 weeks. Healthy subjects attended a single visit, during which medical history taking, 3% hypertonic saline sputum induction, blood sample collection, chest x-ray, and spirometry were performed once only.

***Isolation of sputum supernatant and IL-1β detection***

Spontaneous sputum was collected from patients with bronchiectasis, while 3% hypertonic saline sputum induction was processed only in healthy subjects. After thorough mouth rinsing, patients forcefully expectorated into sterile containers. Sputum plugs were selected after removal of saliva via repeated dragging onto a sterile petri dish. We performed quality-control and split sputum for differential cell counts and bacterial culture. Sputum with a squamous epithelial cell to leucocyte ratio <1:2.5 per low-power microscopic field (magnified ×40) was assayed^[5]^.

We prepared the sputum supernatant by adding four times the volume of phosphate buffer solution (PBS, Corning, USA), followed by 3000 rpm centrifugation for 15 minutes. We detected the expression levels of mature interleukin-1β (IL-1β) (17kDa) in the sputum supernatant of 38 patients with bronchiectasis who had paired stable-AE state samples by using multiplex immunoassay kits according to the reagent instruction (Bio-rad, USA), which have been validated for cytokine measurements.

***Isolation,* in vitro *culture, and stimulation of peripheral blood neutrophils***

We isolated neutrophils from peripheral whole blood samples by using Ficoll-plaque density gradient centrifugation (Cytiva, USA). We diluted the blood samples at a 1:1 ratio with Dulbecco’s Phosphate buffered saline (DPBS, Corning, USA), loaded on a Ficoll layer, and centrifuged for 30 min at room temperature at 800 × g with an acceleration of 1.0 and a deceleration speed of 0. We next discarded the remaining Ficoll solution, while retaining only the bottom layer of the darkish red sediment (primarily containing the erythrocytes, granulocytes, and platelets). We added 30 ml of 1x erythrocyte lysis buffer to each tube and incubated at room temperature, shielded from light, for 10 minutes. We next added EasySep^TM^ Buffer to terminate the lysis reaction, centrifuged at 400 g for 5 minutes, and discarded the supernatant. We washed the sediment with EasySep™ Buffer, centrifuged at 350 g for 5 minutes, and discarded the supernatant. Next, we proceeded with cell counting and adjusted the cell density to 5×10^7 cells/ml. We purified the neutrophils by using an EasySep^TM^ Human Neutrophil Isolation Kit which employs an immunomagnetic negative selection (Stemcell Technologies, USA).

We seeded the freshly isolated neutrophils in a 24-well plate (approximately 5*10^6 cells/well) and cultured in Opti-MEM serum-free medium. We next divided the cells into three groups: the LPS + nigericin stimulation group, the inhibitor intervention group, and the PBS blank control group：

a) We treated the stimulation group with LPS (*P. aeruginosa* PAO1 strain, 500 ng/ml) for 3 hours, followed by continued stimulation with nigericin (10 μM) for 1 hour.

b) In the inhibitor intervention group, we pretreated the cells with the inhibitors Z-VAD-FMK (20 μM), MCC950 (10 μM), GSK484 (10 μM), LDC7559 (10 μM), and disulfiram (10 μM) for 1 hour, followed by stimulation with LPS (*P. aeruginosa* PAO1 strain, 500 ng/ml) for 3 hours and continued the stimulation with nigericin (10 μM) for 1 hour.

c) We treated the blank control group with PBS only.

***Flow cytometric analysis***

For the cells undergoing flow cytometry, we washed them for twice with DPBS and stained with Fixable Viability Stain 510 (BD, USA) in DPBS for 15min at 4℃, followed by incubation with 50 µL of the appropriate antibody-cocktail, including CD45-APC-Cy7 (BD 557833, 1:50, clone 2D1) FITC-CD11b (BD 562793, 1:100, clone ICRF44), PE-CY7-CD15 (BD 560827, 1:100, clone HI98), PerCP/Cyanine 5.5-CD16 (BD 560717, 1:50, clone 3G8), APC-CD66b (BD 561645, 1:50, clone G10F5), and PE-CD62L (BD 555544, 1:100, clone DREG-56)for 30 min at 4℃. Subsequently, we fixed the cells with BD Fix buffer on ice for 20 minutes. We acquired the sample data by using a BD LSR Fortessa (BD, USA), and performed data analysis by using Flow Jo 10.6 (BD, USA).

***Immunofluorescence assays of inflammasome and NETs***

We seeded the neutrophils isolated from bronchiectasis patients on the poly-D-lysine-coated coverslips in 24-well round-bottom culture plates (5.0×10^6^ cells) with 500 µL of Opti-MEM serum-free medium, followed by the addition of the respective stimuli. After removal of the culture medium, we washed the cells for twice with DPBS. We fixed the neutrophils on coverslips with 4% paraformaldehyde for 20 min. We added 300 μl of blocking solution containing 5% bovine serum albumin (BSA), 5% goat serum, and 0.05% Triton X-100, and incubated at room temperature for 30 minutes. We diluted the antibodies of mouse anti-NLRP3 (Adipogen, USA, 1:200), rabbit anti-ASC (Proteintech, USA, 1:300), rabbit anti-citrullinated histone H3 (Abcam, USA, 1:500) and mouse anti-Neutrophil Elastase (Abcam, USA, 1:200) by using the PBS supplemented with 1% BSA, 1% goat serum, and 0.05% Triton X-100. We added 200 μl of the primary antibodies and incubated at 4°C overnight. We washed the slides in PBS at room temperature for three times, each for 5 minutes, and incubated the samples with fluorescent dye-labeled mouse IgG secondary antibody (Alexa Fluor 488, 1:500) and rabbit IgG secondary antibody (Alexa Fluor 555, 1:500) in the dark at room temperature for 1 hour. We removed the antibody solution and incubated the cells in a 5000-fold diluted DAPI solution for 5 minutes. We next washed the slides with PBS twice (5 minutes each time), to remove the unbound secondary antibodies and DNA staining dyes. Finally, we applied 10 μl of the mounting medium onto the slide, gently covered the slide with a coverslip, and utilized the Zeiss confocal LSM 880 microscope to perform confocal microscopy.

***Detection of DNA-NE and DNA-MPO concentration assay***

DNA-NE and DNA-MPO are well-established methodologies, extensively recognized in numerous high-quality peer-reviewed studies, as the most specific, objective, and quantitative techniques for evaluating NETosis^[6]^. After stimulation, we gently aspired the supernatant medium, and gently washed the cells with 500 μl PBS for twice to remove the soluble NE and MPO that was not associated with the NETs. After disruption of the effects of NETs with S7 Nuclease Assay Reagent (15 U/mL; Cayman Chemical, USA) for 15 min at 37℃, we added EDTA (0.5 mmol/L; Cayman Chemical) to stop the reaction. We detected the NET-associated NE by using the NETosis Assay kit (Cayman Chemical)^[7]^. We assayed the NET-associated MPO by using the MPO ELISA kit (Cusabio, Wuhan, China) by following the manufacturer’s instructions. We measured the optical density (OD) value of each replicate well by a microplate reader at 405 nm or 450nm wavelength.

***Western blot***

We precipitated the cell debris by performing centrifugation and collected the soluble proteins in the supernatant. We precipitated the lytic cell protein through chloroform/methanol precipitation via sonication with RIPA containing protease inhibitors and protein from the cell supernatant. We separated an equal amount of protein by performing SDS–PAGE and transferred to the nitrocellulose membranes. We blocked the membranes with 5% skim milk powder for 90 min, followed by incubation with the primary antibodies in 5% skim milk-TBS-T at 4°C overnight with gentle shaking. We added the primary antibodies against IκBα (CST, 1:1000), phosphor-IκBα (CST, 1:1000), NF-κB p65 (CST, 1:2000), phospho-NF-κB p65 (CST, 1:2000), NLRP3 (CST, 1:1000), ASC (Proteintech, 1:1000) IL-1β (CST, 1:1000), Cleaved-IL-1β (CST, 1:1000), Gasdermin D (CST, 1:1000), cleaved N-terminal GSDMD (abcam, 1:1000), Caspase-1(abcam, 1:1000), PAD4 (abcam, 1:1000), β-Actin (CST, 1:2000) and GAPDH (CST, 1:2000). After rinsing with TBS-T for three times, we incubated the membrane with the horseradish peroxidase-conjugated anti-rabbit or mouse secondary antibody (CST, 1:2000) in 5% skim milk-TBS-T. We next visualized the respective protein expression by using a chemiluminescence assay (Tanon 5200, China). We finally analyzed the western blot protein bands for grayscale values by using Image J software.

***Cultures and stimulation of airway epithelial cells at the air-liquid interface***

We grew the basal epithelial cells from bronchial epithelial tissues obtained from patients with bronchiectasis in air-liquid interface (ALI) culture at 37°C by using PneumaCult-Ex Medium (Stemcell, USA) for proliferation and PneumaCult-ALI Medium (Stemcell, USA) for differentiation for cell culture, according to the manufacturer’s instructions.

After a differentiation culture period of 28-30 days, we differentiated the airway epithelial cell into a mature pseudostratified ciliated columnar epithelium at the air-liquid interface, consisting of ciliated cells, goblet cells, and basal cells. We discarded the medium, and washed the differentiated and mature airway epithelial cells for twice with DPBS, and then added the appropriate stimuli.

a) LPS stimulation group: We stimulated with 20 μg/ml LPS (*P. aeruginosa* PAO1 strain) for 24 hours.

b) LPS+ATP stimulation group: We initially stimulated with 20 μg/ml LPS for 24 hours, followed by the addition of 5 mM ATP for an additional 1-hour stimulation.

c) The NETs stimulation group: We collected the peripheral blood from 9 patients with bronchiectasis to isolate the neutrophils. We seeded the freshly isolated neutrophils in a 24-well plate (approximately 5*10^6 cells/well) and cultured by using the Opti-MEM serum-free medium. After 3 hours of stimulation with LPS (*PAO1*, 500 ng/ml), we added nigericin (10 μM) for an additional 1 hour to induce the NETs formation. We next aspired the culture medium, and gently washed the sticky surface of NETs for twice with PBS, with the washed solution being discarded. We scraped the NETs layer from the culture dish and collected in a centrifuge tube, followed by vigorous mixing. We centrifuged the sample at 450 g for 10 minutes at 22°C, and collected the supernatant. Next, we measured the dsDNA concentration by using the Quant-iT^TM^ PicoGreen^TM^ dsDNA Kit (Thermo Fisher Scientific, USA). We stimulated the airway epithelial cells for 24 hours with the collected NETs solution, which has an equivalent dsDNA concentration of approximately 500 ng/ml.

d) NETs + LPS stimulation group: We stimulated the airway epithelial cells with the medium containing Aeruga LPS (20 μg/ml) and NETs (dsDNA equivalent concentration approximately 500 ng/ml) for 24 h.

e) We supplemented the blank control group with the basal medium only.

***Quantitative Polymerase Chain Reaction (QPCR)***

Following lysis in TaKaRa MiniBEST Universal RNA Extraction Kit (Taraka, Japan) reagent, we isolated the RNA according to the manufacturer’s protocol. We reverse transcribed the RNA into cDNA through reverse transcription using the PrimeScript^TM^ RT Master Mix Kit (Taraka, Japan). We then measured the relative mRNA RNA expression levels by using the TB Green^®^ Premix Ex Taq^TM^ II (Taraka, Japan) on the Quantstudio 7 Real Time PCR System (Applied Biosystems, USA).

Blow is the list regarding the synthesized primers

| **Target gene** | **Forward primer(5’-3’)** | **Reverse primer(5’-3’)** |
| --- | --- | --- |
| NLRP3 | AAGGGCCATGGACTATTTCC | GACTCCACCCGATGACAGTT |
| ASC | AAGCCAGGCCTGCACTTTAT | CTGGTACTGCTCATCCGTCA |
| Caspase-1 | CCACAATGGGCTCTGTTTTT | CATCTGGCTGCTCAAATGAA |
| GSDMD | GTGTGTCAACCTGTCTATCAAGG | CATGGCATCGTAGAAGTGGAAG |
| IL-1β | CTGAAAGCTCTCCACCTCCA | CCAAGGCCACAGGTATTTTG |
| IL-18 | CAACAAACTAGTTTGTCGCAGGA | TGCCACAAAGTTGATGCAAT |
| IL-1α | TGTATGTGACTGCCCAAGATGAAG | AGAGGAGGTTGGTCTCACTACC |
| IL-6 | CGGGAACGAAAGAGAAGCTCTA | CGCTTGTGGAGAAGGAGTTCA |
| IL-8 | CTTGGTTTCTCCTTTATTTCTA | GCACAAATATTTGATGCTTAA |
| TNF-α | AACATCCAACCTTCCCAAACG | GACCCTAAGCCCCCAATTCTC |
| CXCL-1 | AGCTTGCCTCAATCCTGCATCC | TCCTTCAGGAACAGCCACCAGT |
| CXCL2 | GGCAGAAAGCTTGTCTCAACCC | CTCCTTCAGGAACAGCCACCAA |
| CXCL5 | CAGACCACGCAAGGAGTTCATC | TTCCTTCCCGTTCTTCAGGGAG |
| CXCL6 | GGGAAGCAAGTTTGTCTGGACC | AAACTGCTCCGCTGAAGACTGG |
| GAPDH | GAAGGTGAAGGTCGGAGTC | GAAGATGGTGATGGGATTTC |

All synthesis was achieved by Sangon Biotechnology (Shanghai, China).

***RNA sequencing***

The construction of the cDNA library adhered to the established protocol of the Hieff NGS^®^ Ultima Dual-mode mRNA Library Prep Kit for Illumina® (Yeasen Biotechnology (Shanghai) Co., Ltd China). We conducted sequencing of the cDNA libraries by using the Illumina NovaSeq 6000 platform (Gene Denovo Biotechnology (Guangzhou) Co., Ltd China). We aligned the sequencing reads to the human reference genome (Ensembl release 106) and quantified for each gene based on the RSEM algorithm. We did gene expression normalization by using EDASEQ, and conducted differential gene expression analysis by using DESeq2 (version 1.10.1) with a significance threshold of Q-value < 0.05 and log_2_(fold-change) > 1. We selected the differentially expressed mRNA for GO and KEGG pathway analysis.

***Histology and Immunocytochemistry***

After washing with PBS, we fixed the differentiated airway epithelial cells in 4% neutral buffered formalin and embedded in paraffin, sectioned (5 µm; microtome), mounted and stained with hematoxylin and eosin.

For immunofluorescence assays, we fixed Transwell membranes from ALI cultures by using cold (-20°C) methanol for overnight incubation at -20°C and cold acetone (-20°C) treatment for 1 minute. After washing by PBS, we then blocked the cells with blocking buffer (PBS, 2 % goat serum, 1% BSA, 0.1% Cold fish skin gelatin, 0.1% Triton^TM^ X-100, 0.05% TWEEN^®^ 20) at room temperature for 1 hour. We then washed the cells with primary antibody overnight at 4°C. We applied the primary antibodies of MUC5AC (1:50, Thermo Fisher Scientific, USA), ZO-1 (1:100, CST, USA) and acetylated tubulin (1:100, Abcam, USA) in PBS (1% BSA, 0.1% Cold fish skin gelatin). We rinsed the Transwell membranes for three times with DPBS, stained with appropriate species-specific secondary antibody diluted at 1:500 included goat anti-Mouse IgG (H+L), Alexa Fluor 488 and goat anti-rabbit IgG (H+L), Alaxa-555 (Thermo Fisher Scientific, USA) for 2 hrs. We washed the cells for three times with PBS, stained with the nuclear stain 4’,6-diamidino-2-phenylindole (DAPI, Thermo Fisher Scientific, USA) for 10 min at RT. We next excised and mounted the transwell membranes on the glass slides with ProLong Gold Antifade Mountant (Thermo Fisher Scientific, USA). We randomly selected three to five areas (×400 magnification) at the ALI culture surface and imaged by using the Zeiss LSM 880 confocal microscope (ZEISS, Germany) at a 40× or 20× oil immersion objective.

***Cytokine release assay***

We assayed the human IL-1β concentrations in cell-culture supernatants with an ELISA kit (RD, USA), according to the manufacturer's protocols.

***Statistical analysis***

We performed statistical analysis by using GraphPad Prism 10.0 (Graphpad Inc., San Diego, USA) and SPSS 26 (SPSS Inc., Chicago, USA). We evaluated the normality of continuous variables by using the Kolmogorov-Smirnov test. We presented the normally distributed continuous variables as mean ± standard deviation, while skewed variables as median (interquartile range). We analyzed the correlation of continuous variables by using Pearson’s correlation model. We compared the continuous variables between groups by using the independent-sample t-test, paired t-test, or non-parametric tests based on their normal distribution intervals. We presented the categorical variab as the frequency and percentage, and analyzed the differences between groups by using chi-square test or Fisher’s Exact test. ROC curves were adopted to display the diagnostic value of sputum IL-1β and blood neutrophil percentages in distinguishing patients with AE from those in a stable state, with the calculation of the area under the curve and the corresponding 95% confidence interval.

**Supplementary Reference**

1. Aliberti S, Goeminne PC, O'Donnell AE, et al. Criteria and definitions for the radiological and clinical diagnosis of bronchiectasis in adults for use in clinical trials: international consensus recommendations. *The Lancet Respiratory Medicine* 2022: 10(3): 298-306.

2. Hill AT, Haworth CS, Aliberti S, et al. Pulmonary exacerbation in adults with bronchiectasis: a consensus definition for clinical research. *European Respiratory Journal* 2017: 49(6).

3. Reiff DB, Wells AU, Carr DH, et al. CT findings in bronchiectasis: limited value in distinguishing between idiopathic and specific types. *AJR Am J Roentgenol* 1995: 165(2): 261-267.

4. Chalmers JD, Goeminne P, Aliberti S, et al. The bronchiectasis severity index. An international derivation and validation study. *Am J Respir Crit Care Med* 2014: 189(5): 576-585.

5. Gao Y-h, Guan W-j, Xu G, et al. The Role of Viral Infection in Pulmonary Exacerbations of Bronchiectasis in Adults. *Chest* 2015: 147(6): 1635-1643.

6. Masuda S, Nakazawa D, Shida H, et al. NETosis markers: Quest for specific, objective, and quantitative markers. *Clinica Chimica Acta* 2016: 459: 89-93.

7. Zhang H, Qiu SL, Tang QY, et al. Erythromycin suppresses neutrophil extracellular traps in smoking-related chronic pulmonary inflammation. *Cell Death Dis* 2019: 10(9): 678.

**Supplementary Tables**

**Supplementary Table S1.** Age, gender and the location of bronchial biopsy among the four bronchiectasis patients

|  | Age | Gender | The bronchiectatic lobe |
| --- | --- | --- | --- |
| Bronchiectasis Patient No.1 | 42 | Female | Right middle lobe |
| Bronchiectasis Patient No.2 | 46 | Male | Right lower lobe |
| Bronchiectasis Patient No.3 | 34 | Female | Left lower lobe |
| Bronchiectasis Patient No.4 | 54 | Male | Right lower lobe |

None of the patients had received inhaled antibiotics before bronchial biopsy.

None of the patients was deemed to have bronchiectasis-chronic obstructive pulmonary disease overlap.

**Supplementary figure legends**

**Supplementary Figure S1. Expression of surface markers on neutrophils in patients with bronchiectasis.**

(A) Gating Strategy for Flow Cytometry.

(B) and (C) Comparison of expression levels of neutrophil surface markers among different patient groups.

Groups are compared by using the paired t-test.

**Supplementary Figure S2.** **Comparison of NLRP3, ASC and caspase-1 p20** **protein expression levels between the healthy controls group and the bronchiectasis group**

(A) Western blot analyses of NLRP3, ASC, caspase-1 p20 and endogenous control GAPDH in blood neutrophils from patients with bronchiectasis (BE) and healthy individuals (HC) with LPS+nigericin costimulation (three representative samples each at BE and HC).

(B) Comparison of NLRP3, ASC and caspase-1 p20 protein expression levels between the healthy individuals and the patient group (n = 3 per group). Groups were compared using independent t tests.

BE: bronchiectasis; HC: healthy individuals

**Supplementary Figure S3. Comparison of NLRP3 and ASC levels in blood neutrophils between the healthy individuals and the patient group**

1. and (C) Immunofluorescence staining of NLRP3 (green), ASC (red) and DAPI (blue) in neutrophils (200X, scale bar = 50 μm).

(B) Comparison of NLRP3 and ASC fluorescence intensity levels between the healthy individuals and the bronchiectasis patient group under unstimulated conditions (n = 3 per group, data were obtained from 5 randomly selected fields per group).

(D) Comparison of NLRP3 and ASC fluorescence intensity levels between the healthy individuals and the patient group after stimulated (n = 3 per group, data were obtained from 5 randomly selected fields per group).

BE: bronchiectasis; HC: healthy individuals

**Supplementary Figure S4.** **Comparison of NLRP3 and ASC levels between the stable group and the AE group without stimulation**

（A）Western blot analyses of NLRP3, ASC and endogenous control GAPDH in blood neutrophils from patients with bronchiectasis (three representative samples each at stable state and AE).

(B) Comparison of NLRP3 and ASC protein expression levels between the stable group and the AE group without stimulation (n = 6 each group).

AE: acute exacerbation

**Supplementary Figure S5. Comparison of NETs levels in blood neutrophils between the healthy individuals and the patient group**

(A) Immunofluorescence staining of NE (green), CitH3 (red) and DAPI (blue) in neutrophils (200X, scale bar = 50 μm).

(B) Comparison of NETs-positive area between the healthy individuals and the bronchiectasis patients during AE (n = 3 per group, data were obtained from 5 randomly selected fields per group)..

NETs: neutrophil extracellular traps; CitH3: citrullinated histone H3; NE: neutrophil elastase; AE: acute exacerbation; HC: healthy individuals

**Supplementary Figure S6. Comparison of NLRP3 inflammasome marker mRNA levels between the control and the LPS groups (n = 3 each group).**

Groups are compared by using the paired t-test.

**Supplementary Figure S7. LPS-induced activation of the NLRP3 inflammasome pathway in airway epithelial cells of** **healthy individuals.**

(A) Western blot analyses of NLRP3, caspase-1, pro-IL-1β, cleaved-caspase-1 and the endogenous control GAPDH in bronchial epithelial cells (n = 3 per group).

(B) Comparison of the protein expression levels of NLRP3, caspase-1 and pro-IL-1β between the control group and the LPS + ATP group (n = 3 per group).

**Supplementary Figure S8.** **The RNA sequencing analysis reveals differential gene expression between the LPS-treated group and the control group.**

The analysis reveals differentially expressed genes between the LPS group and the control group, as demonstrated by the heatmap (A) and volcano plot (B).

(C) The LPS group exhibit up-regulation of 143 genes and down-regulation of 58 genes compared with the control group.
