# Supplementary figures and images for: NLRP3 Inflammasome Activation Modulates Neutrophil Extracellular Trap Formation and Aggravates Airway Inflammation in Bronchiectasis
Source: Research (Wash D C). 2025 Oct 17;8:0958. doi: 10.34133/research.0958 (PMC12696696; doi:10.34133/research.0958)

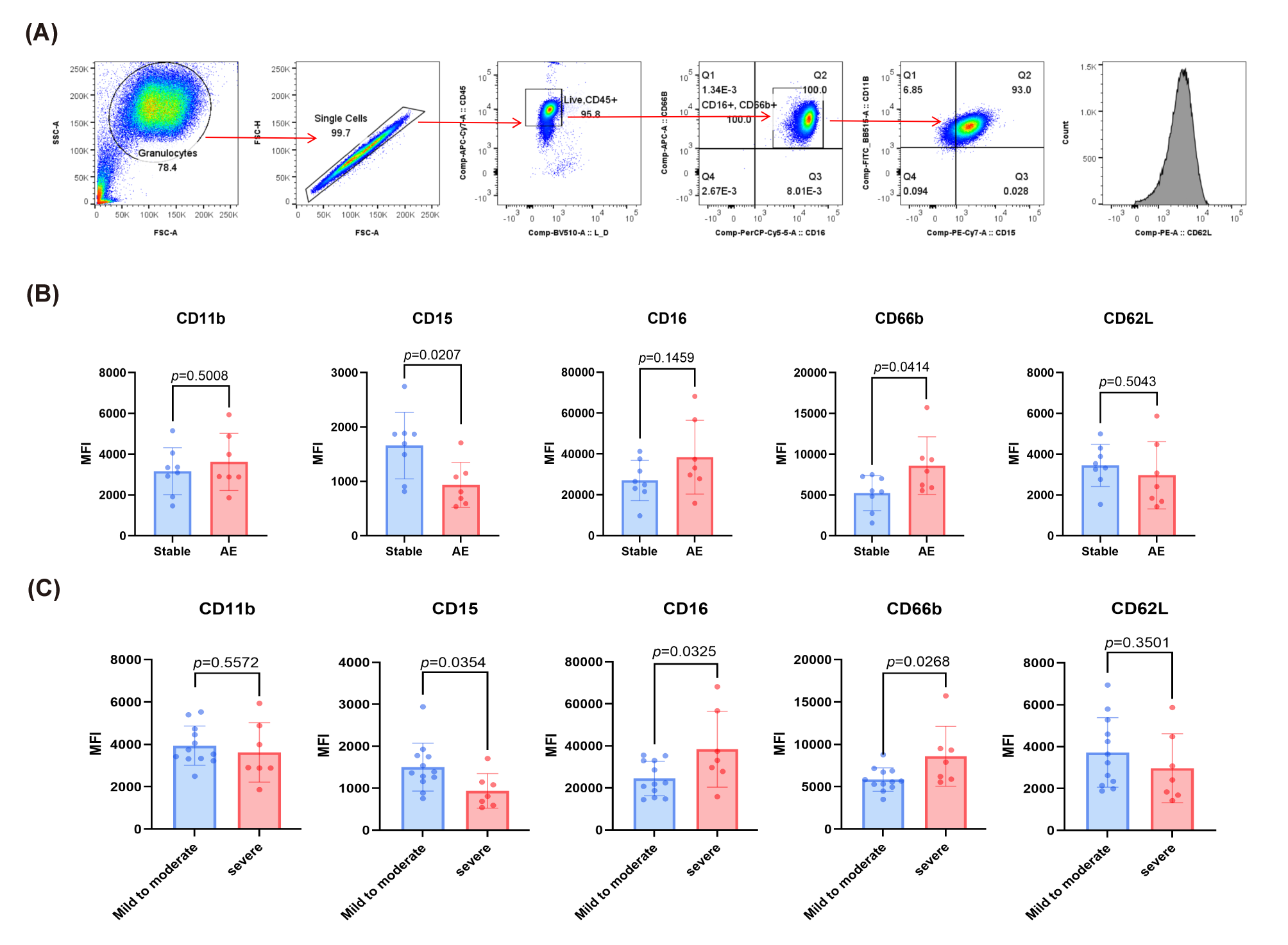

Supplement: Supplementary 1 — Supplementary Methods Figs. S1 to S8 Table S1 [file research.0958.f1.zip › Supplementary FigureS1.tif]

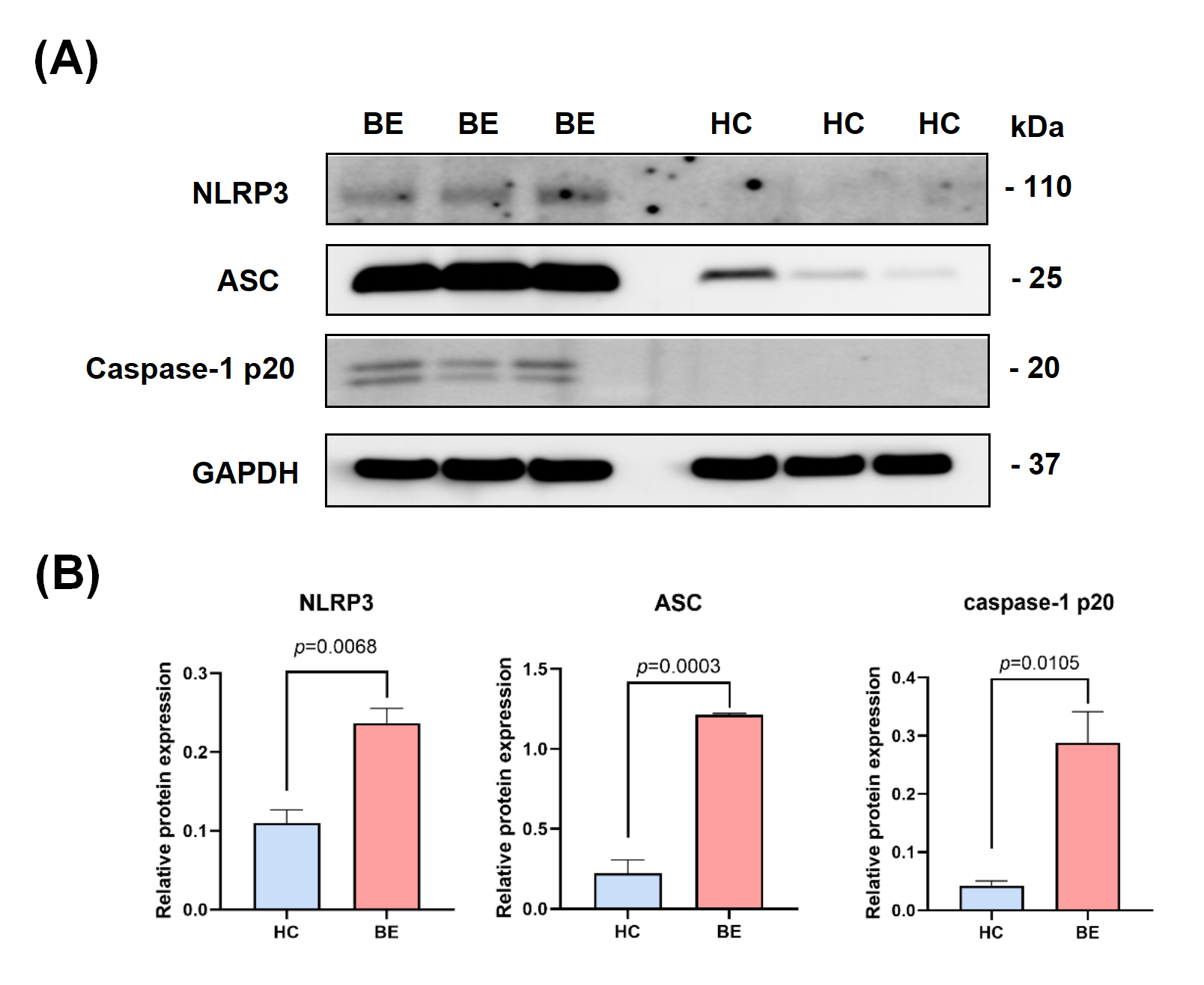

Supplement: Supplementary 1 — Supplementary Methods Figs. S1 to S8 Table S1 [file research.0958.f1.zip › Supplementary FigureS2.tif]

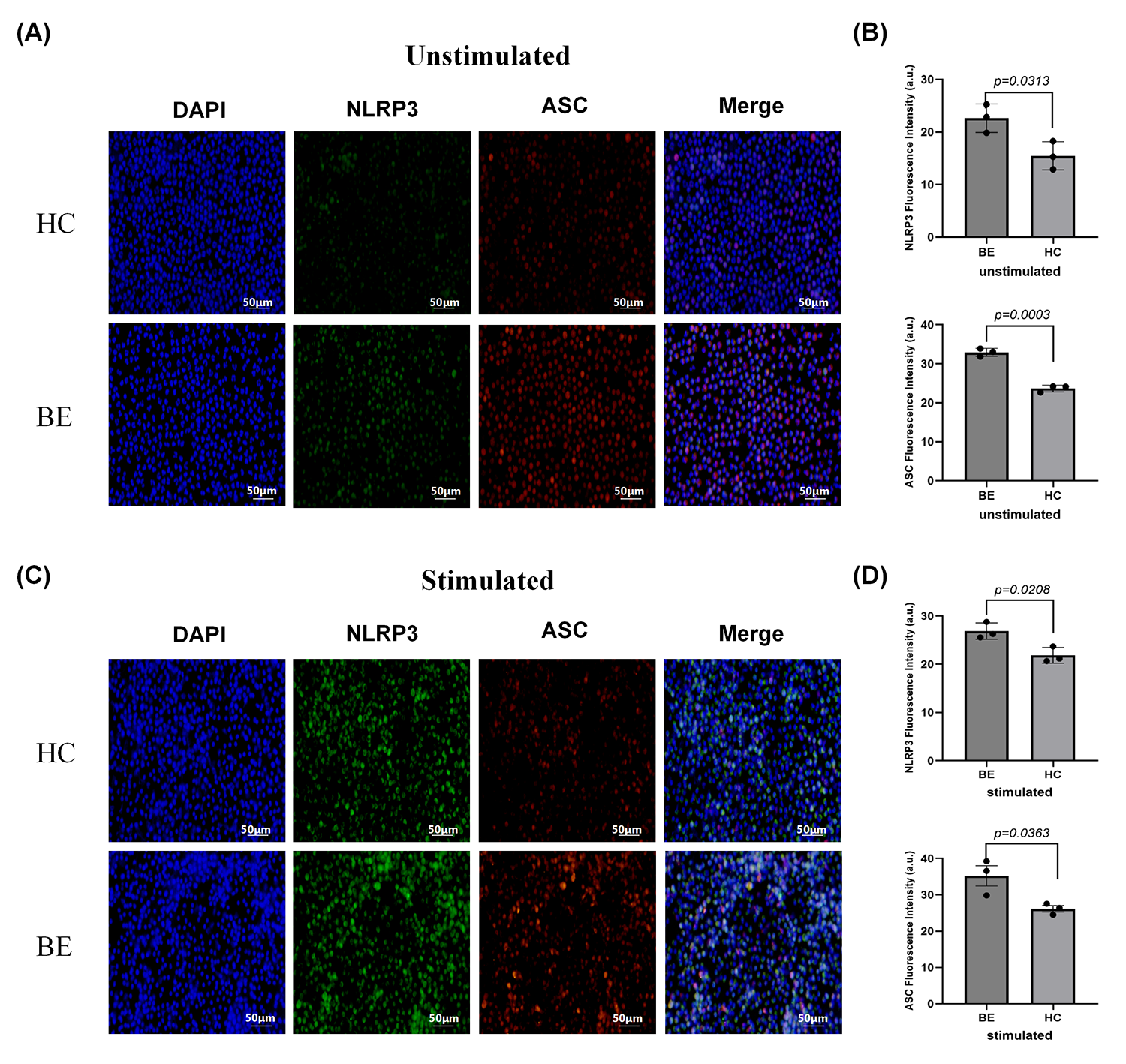

Supplement: Supplementary 1 — Supplementary Methods Figs. S1 to S8 Table S1 [file research.0958.f1.zip › Supplementary FigureS3.tif]

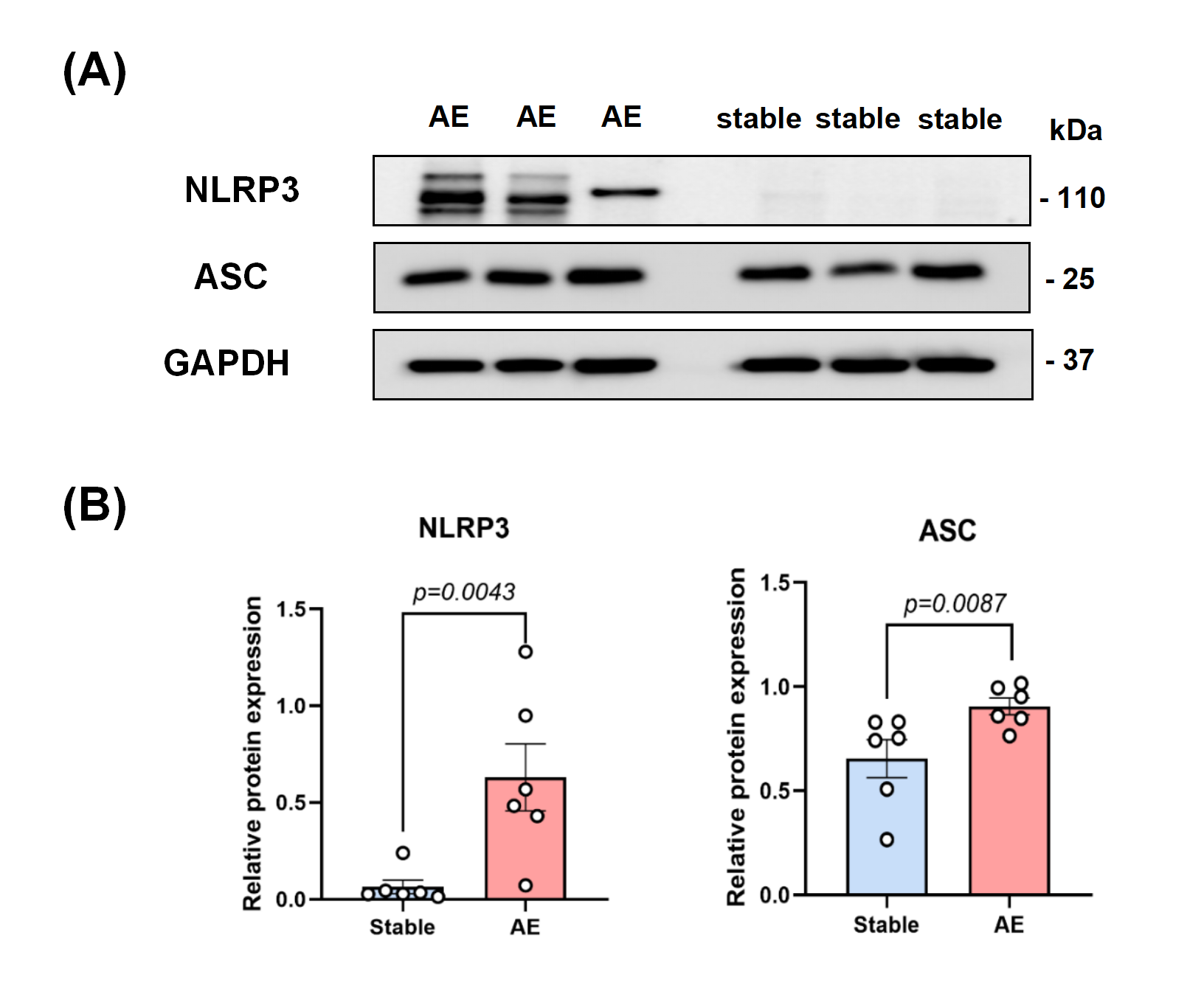

Supplement: Supplementary 1 — Supplementary Methods Figs. S1 to S8 Table S1 [file research.0958.f1.zip › Supplementary FigureS4.tif]

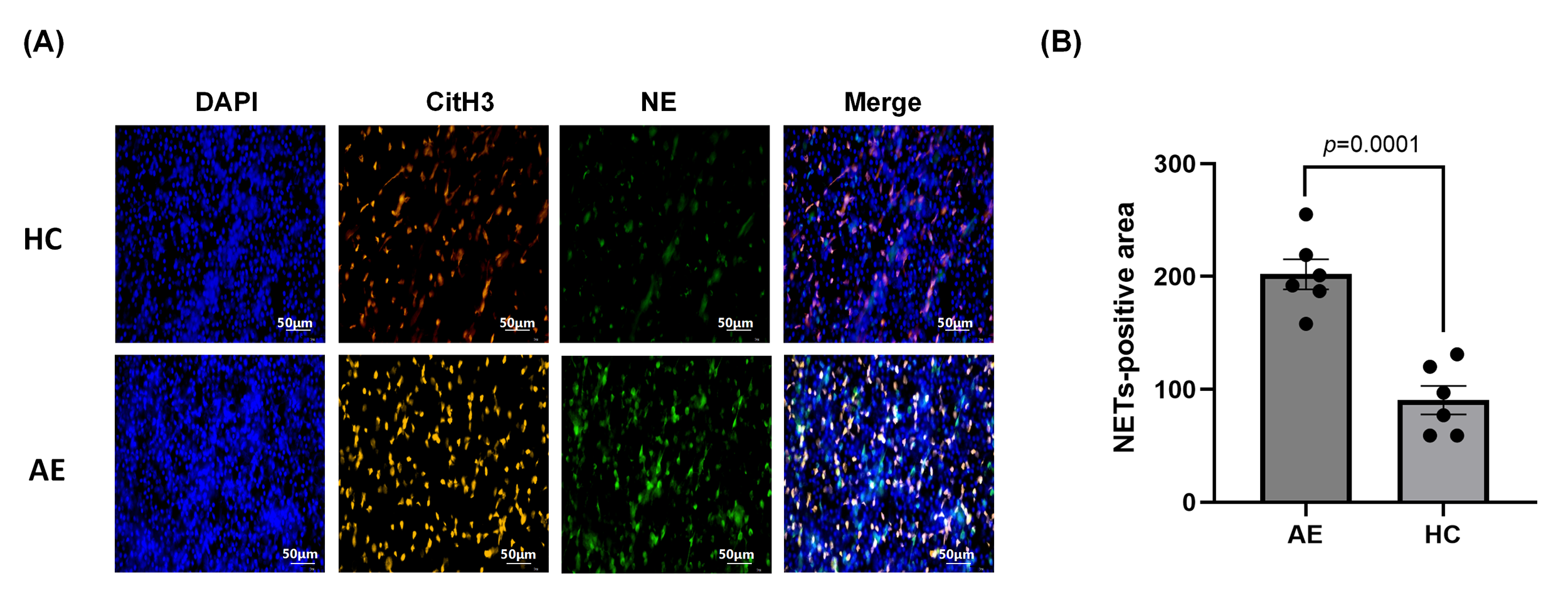

Supplement: Supplementary 1 — Supplementary Methods Figs. S1 to S8 Table S1 [file research.0958.f1.zip › Supplementary FigureS5.tif]

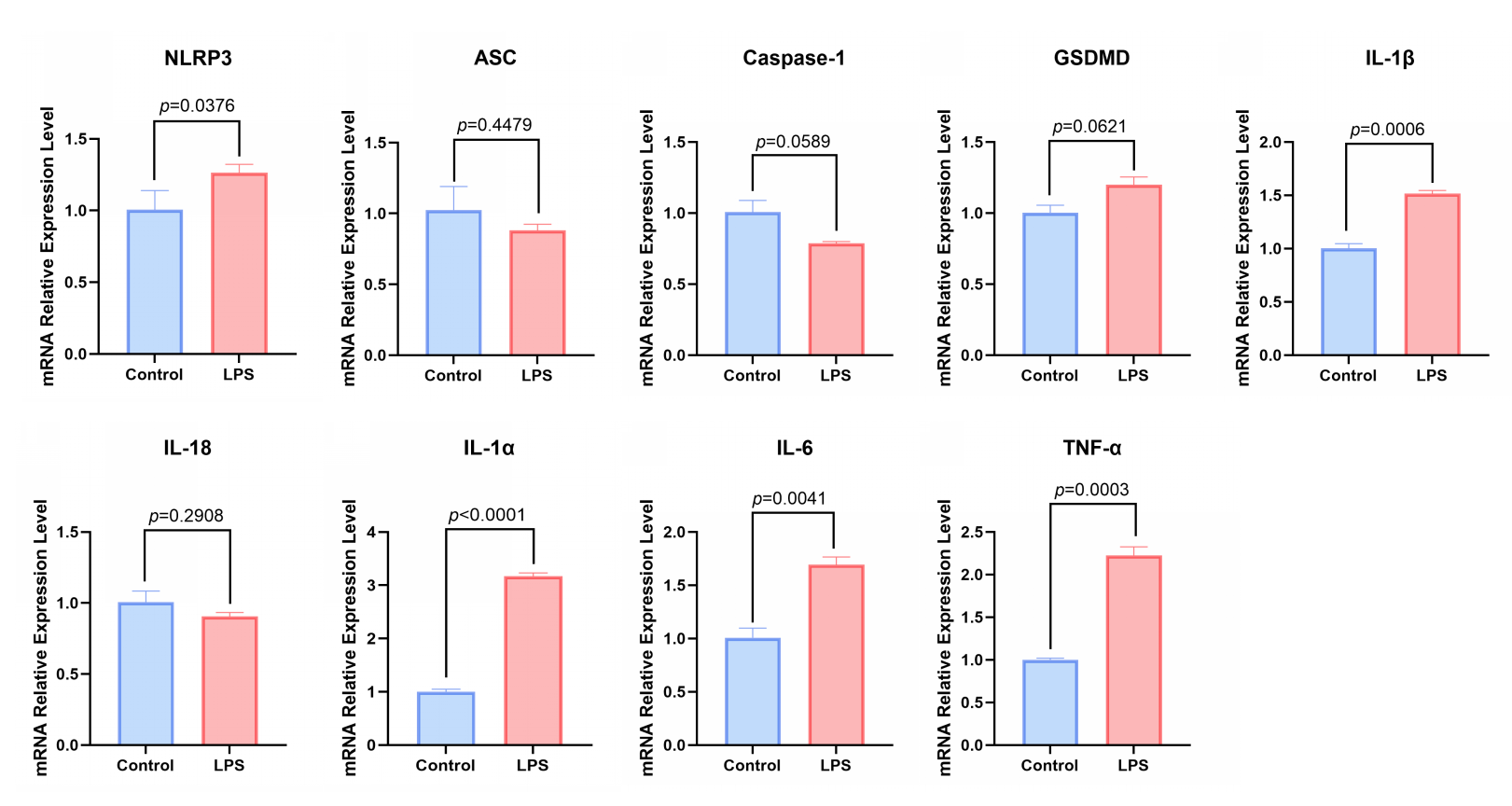

Supplement: Supplementary 1 — Supplementary Methods Figs. S1 to S8 Table S1 [file research.0958.f1.zip › Supplementary FigureS6.tif]

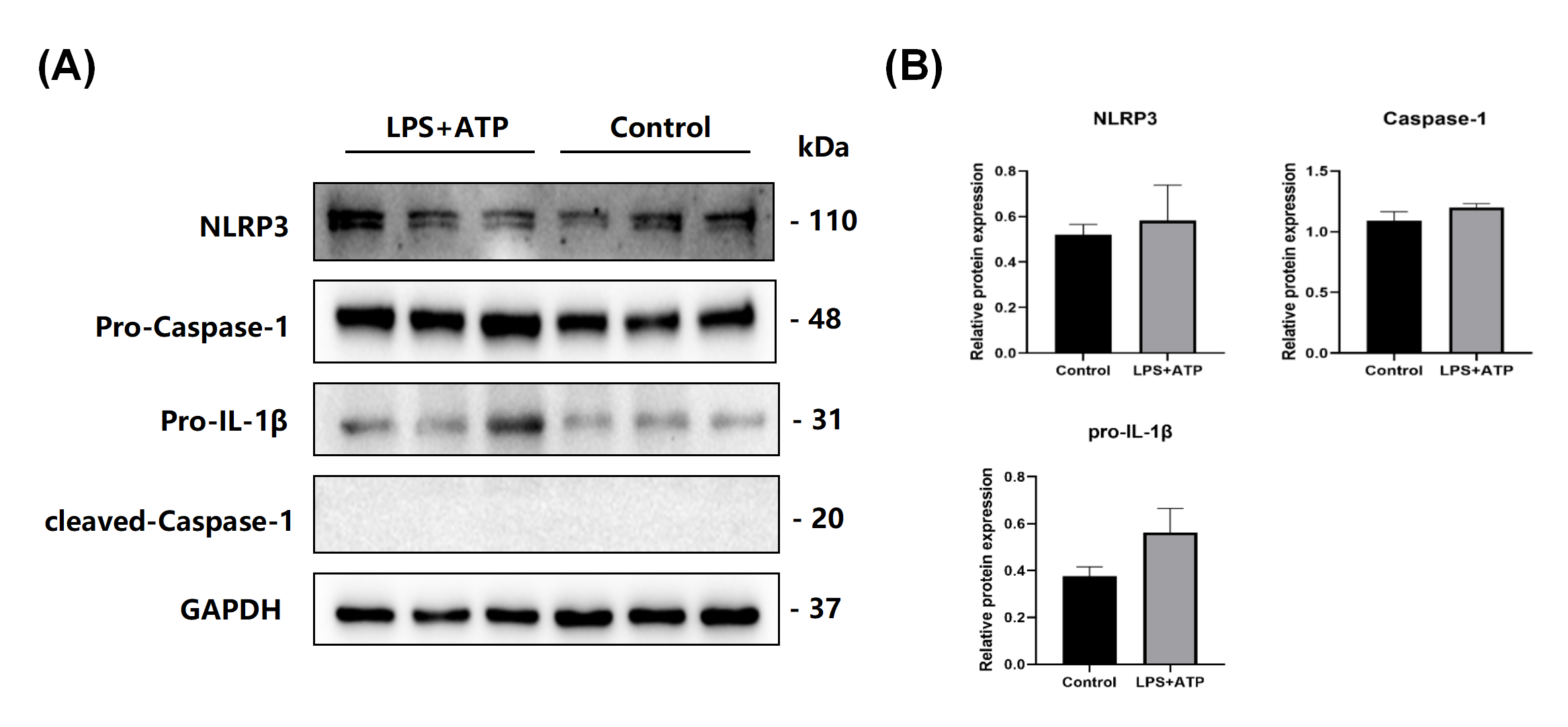

Supplement: Supplementary 1 — Supplementary Methods Figs. S1 to S8 Table S1 [file research.0958.f1.zip › Supplementary FigureS7.tif]

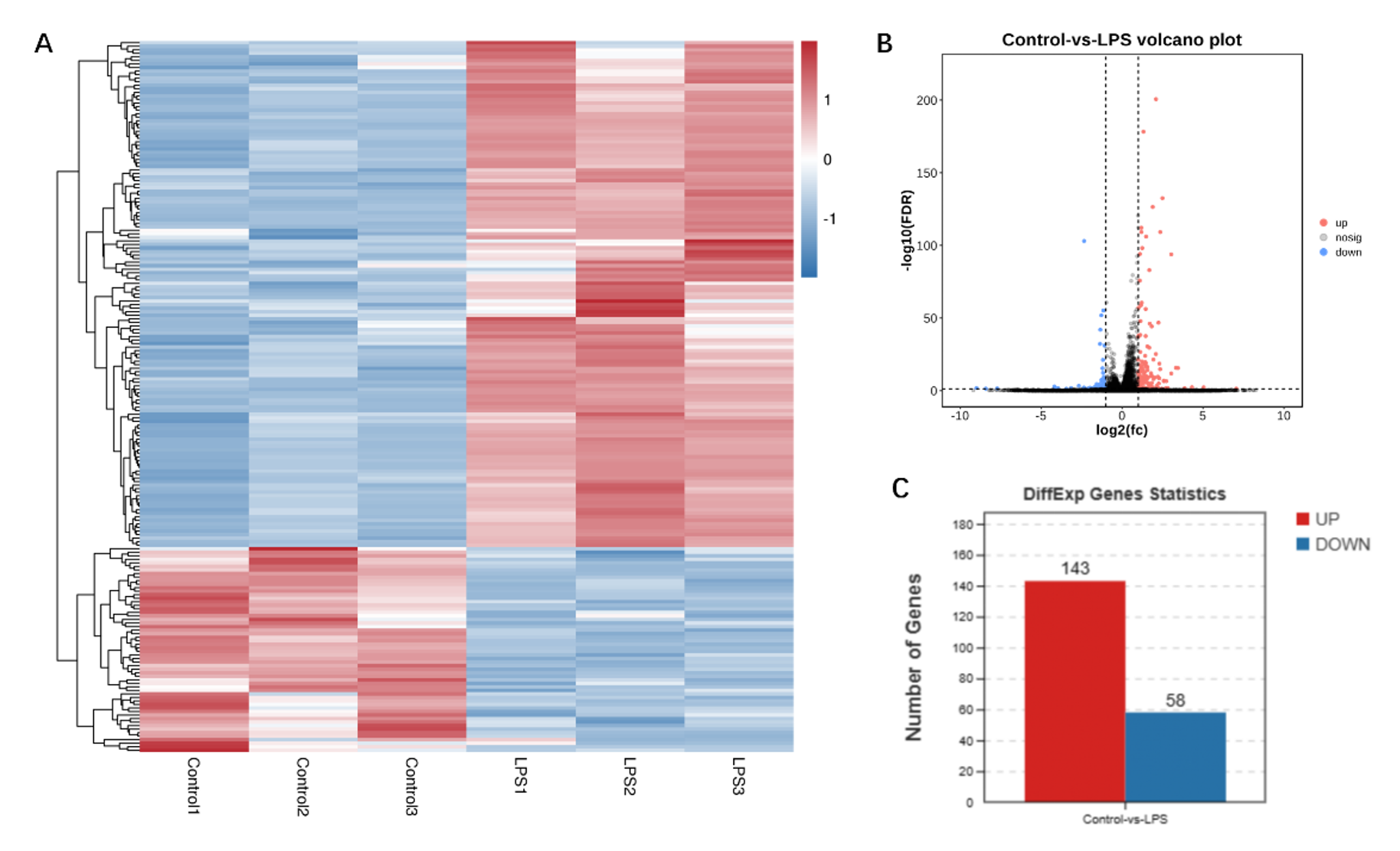

Supplement: Supplementary 1 — Supplementary Methods Figs. S1 to S8 Table S1 [file research.0958.f1.zip › Supplementary FigureS8.tif]
